# Supplementary material for: Inhibition of VEGF receptors induces pituitary apoplexy: An experimental study in mice
Source: PLoS One. 2023 Mar 16;18(3):e0279634. doi: 10.1371/journal.pone.0279634 (PMC10019612; doi:10.1371/journal.pone.0279634)

## Slide 1
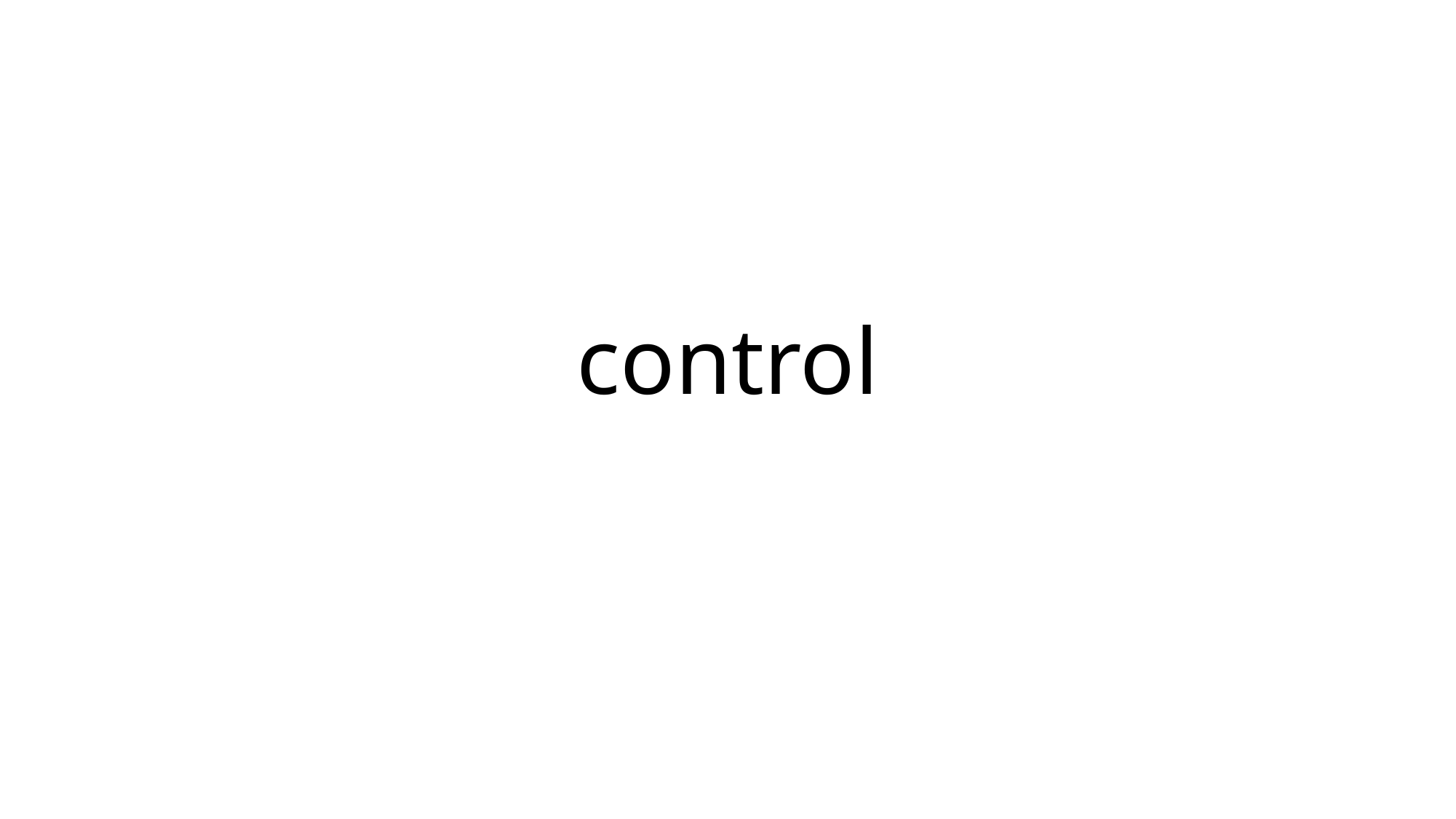

# control

## Slide 2
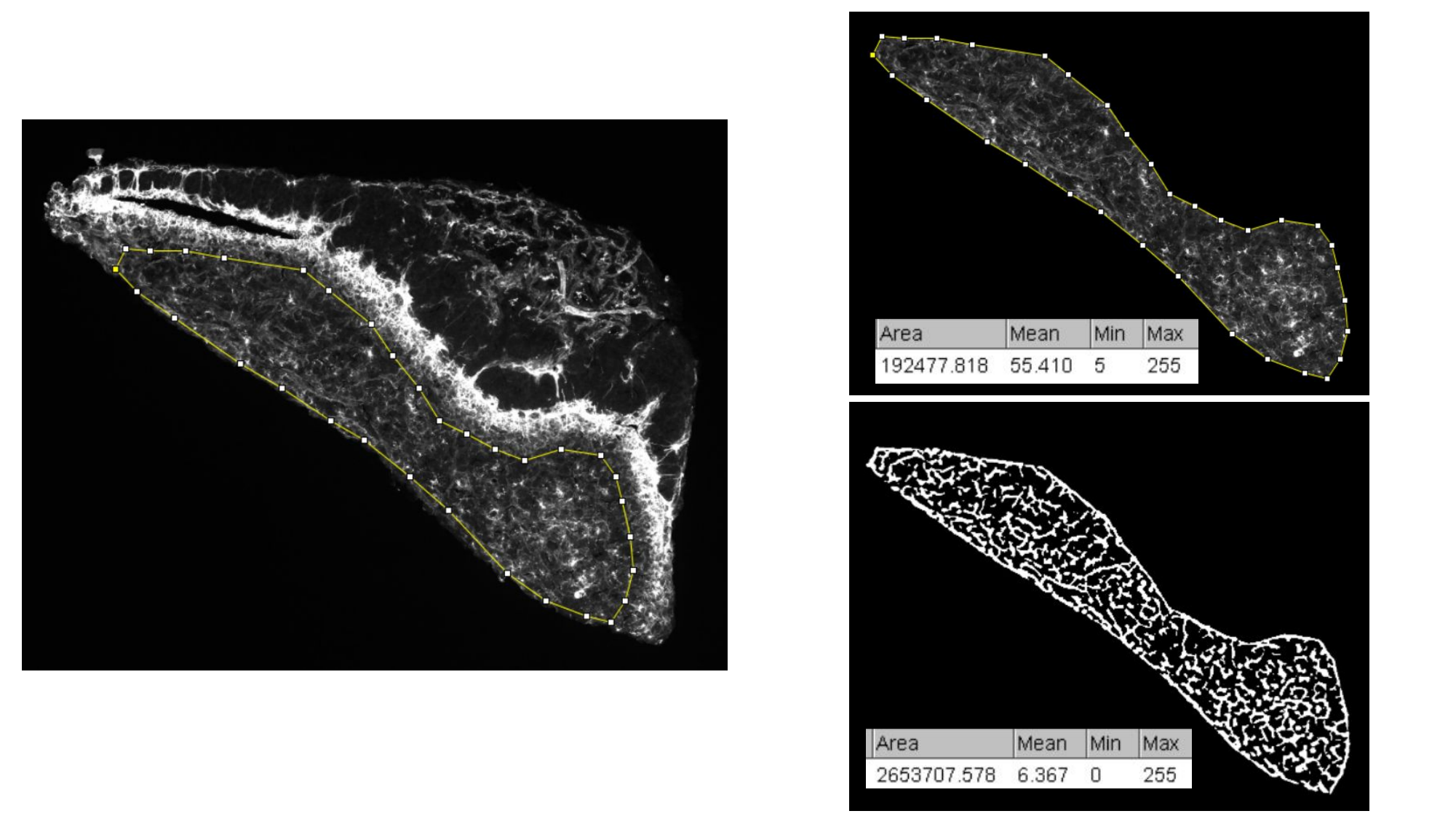

## Slide 3
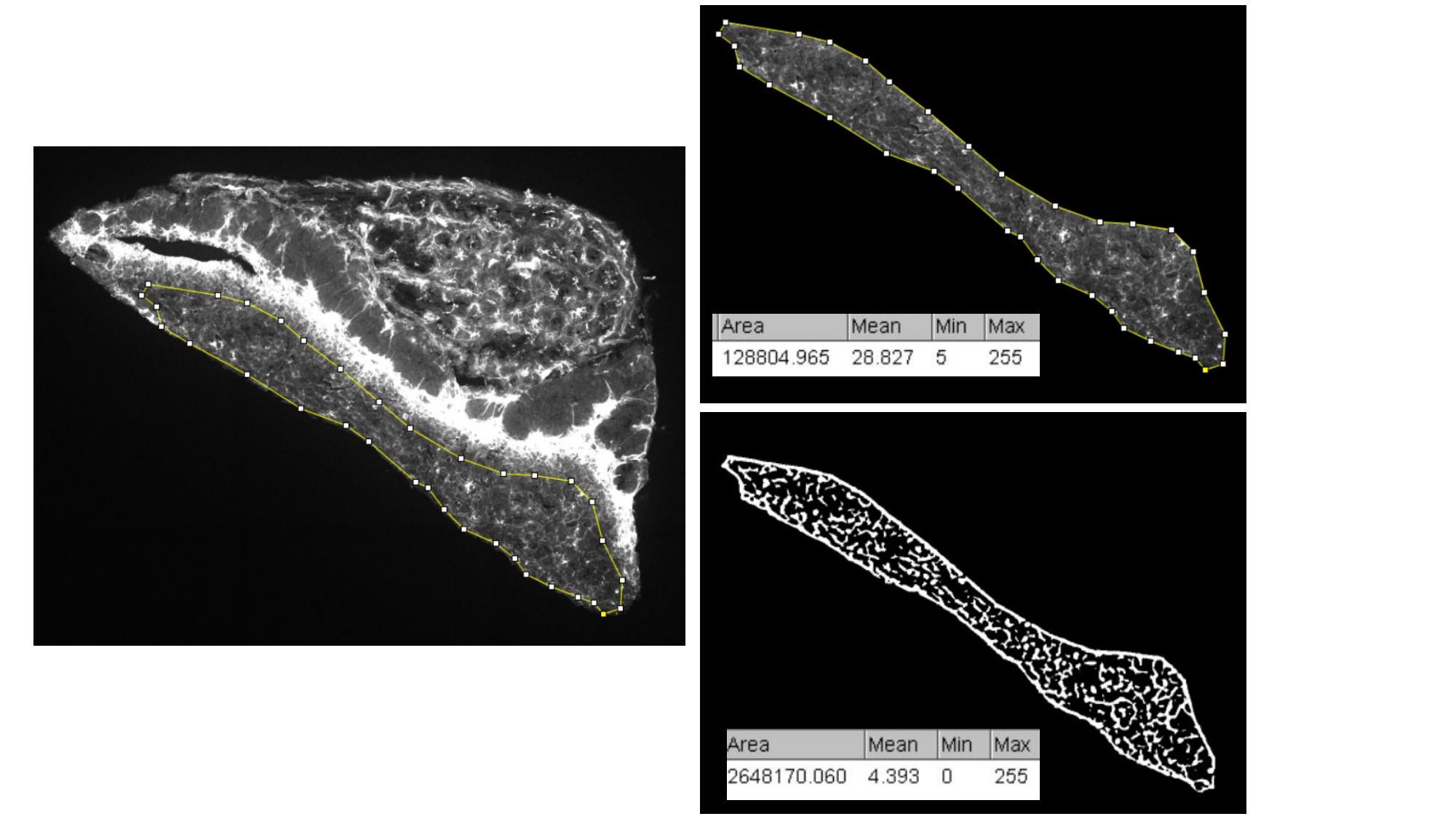

## Slide 4
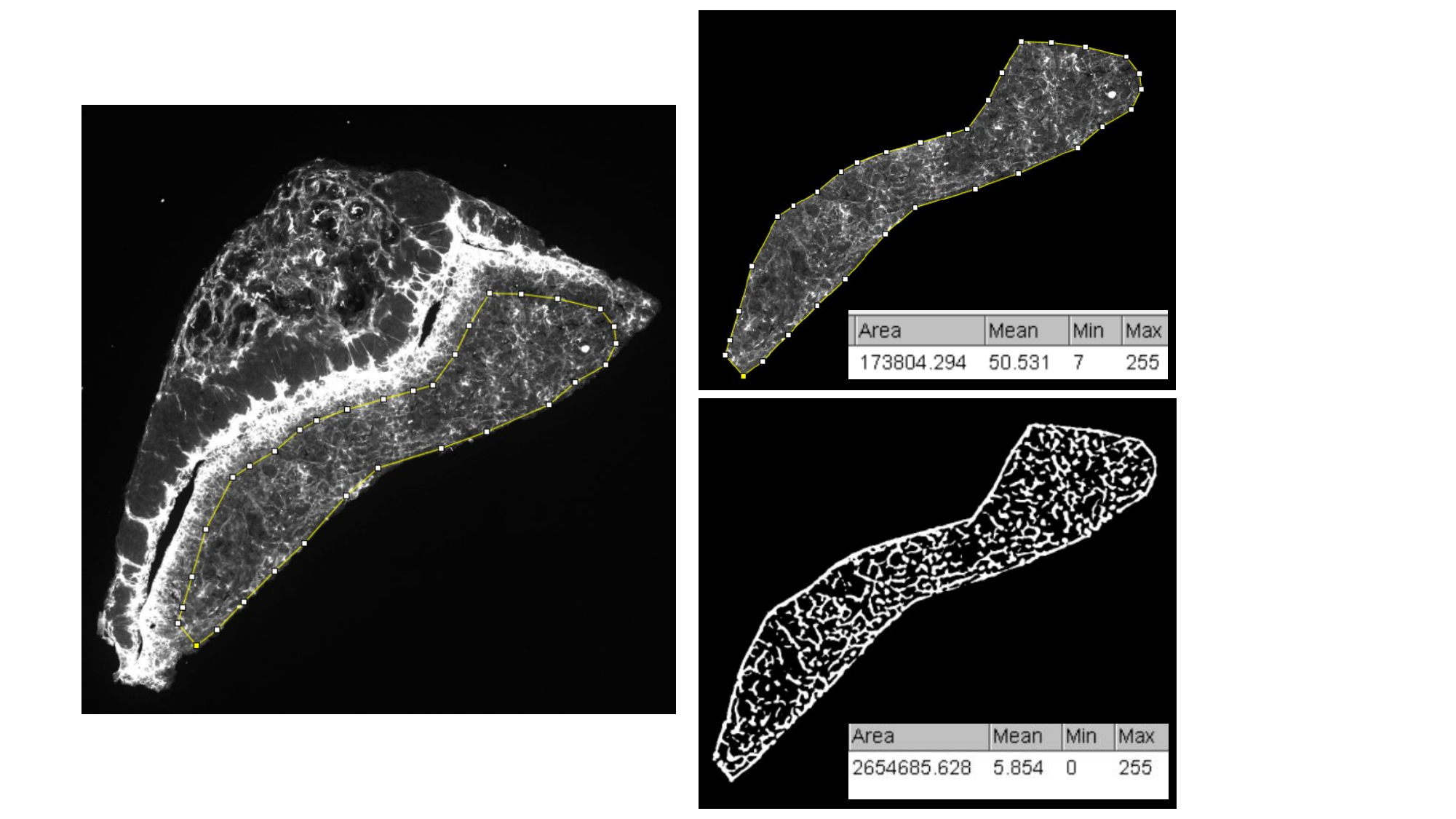

## Slide 5
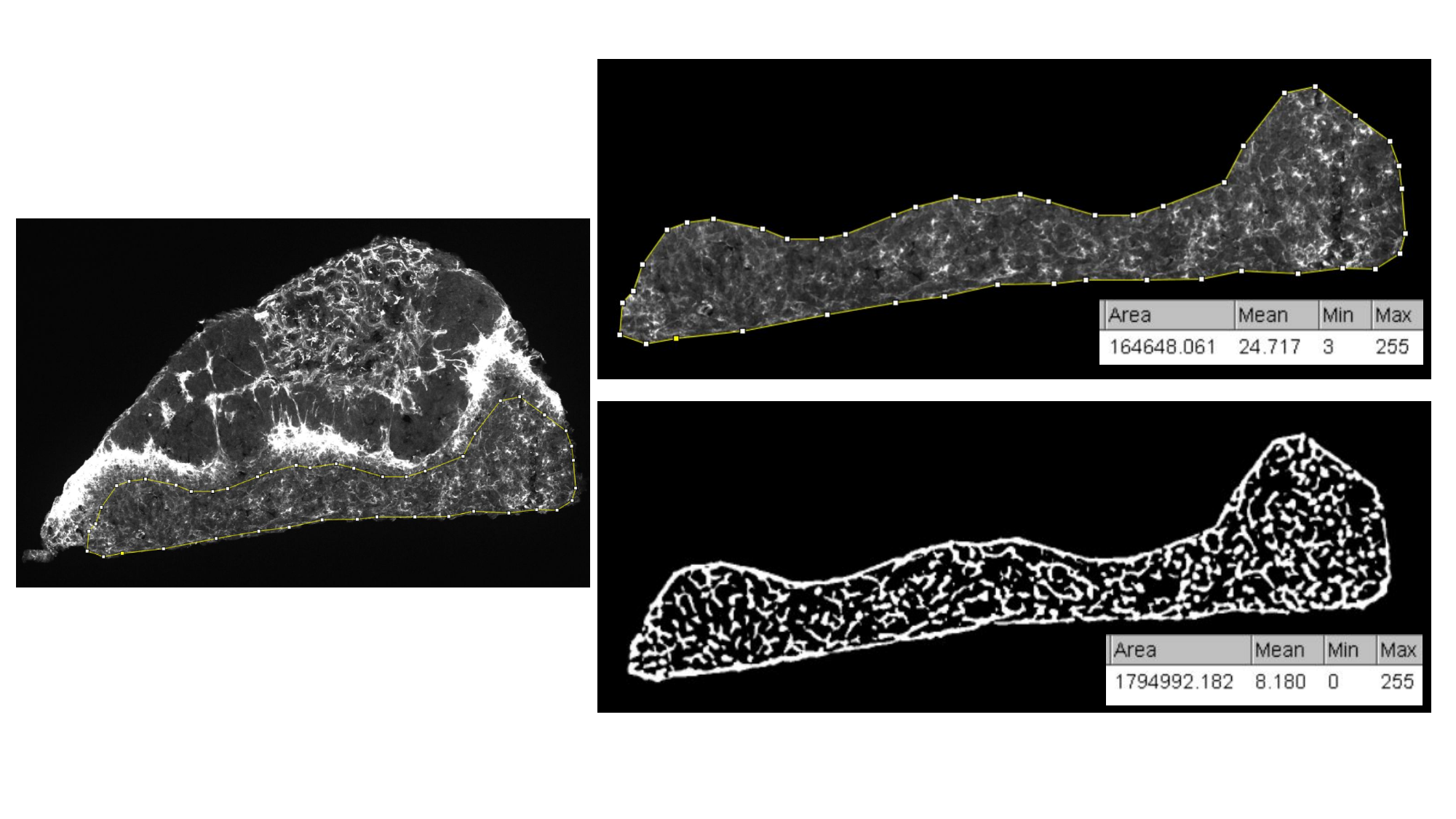

## Slide 6
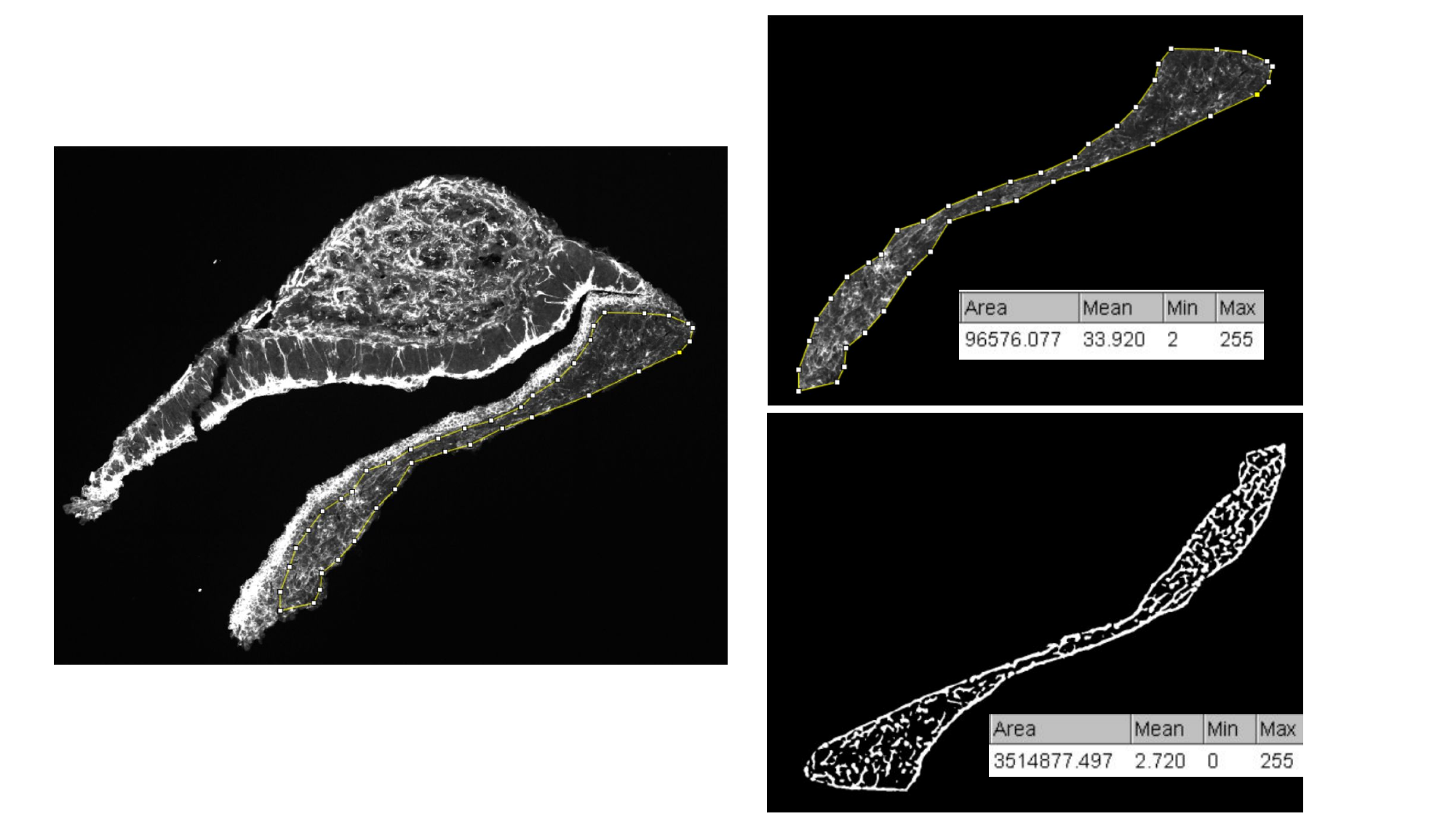

## Slide 7
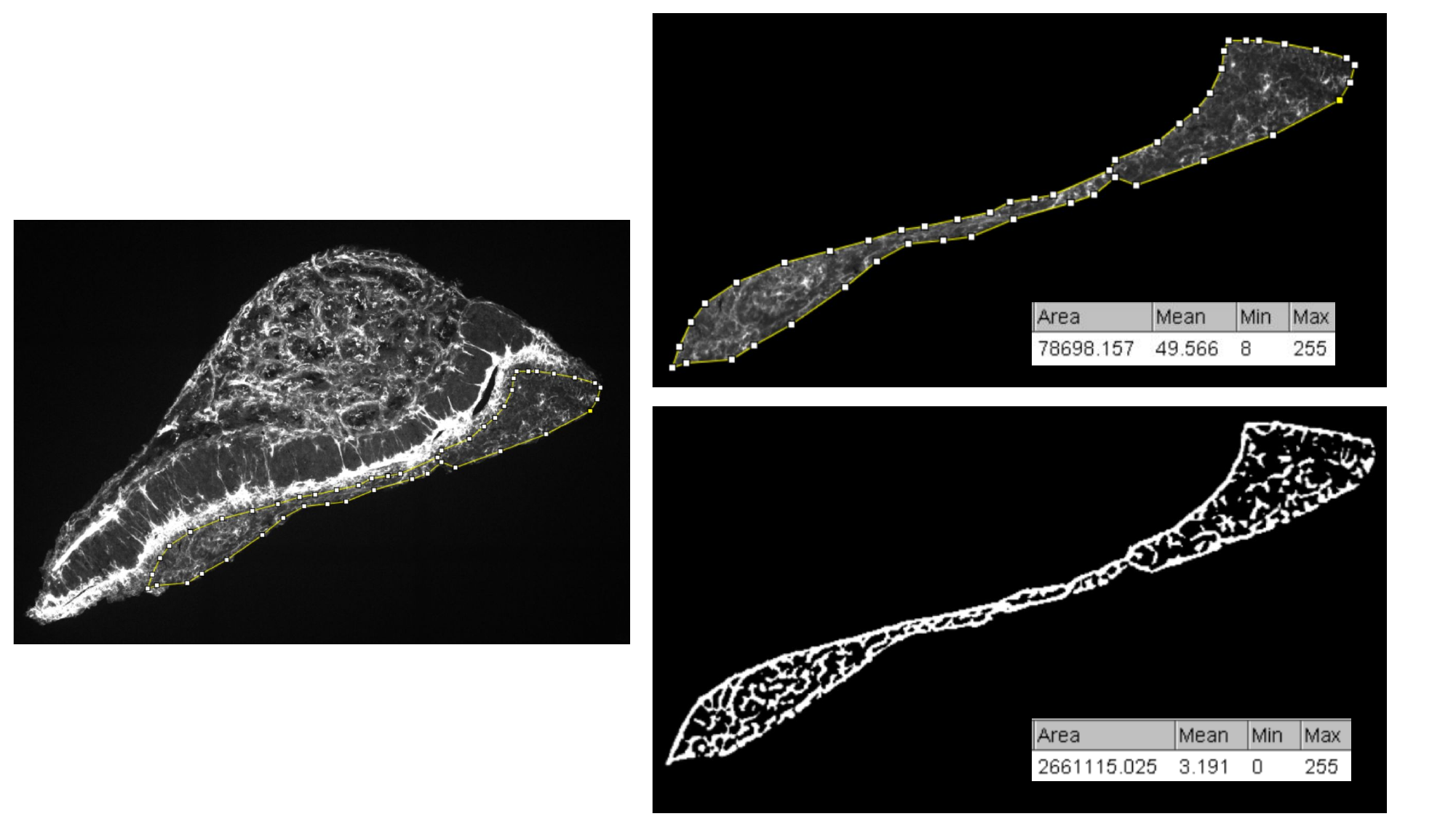

## Slide 8
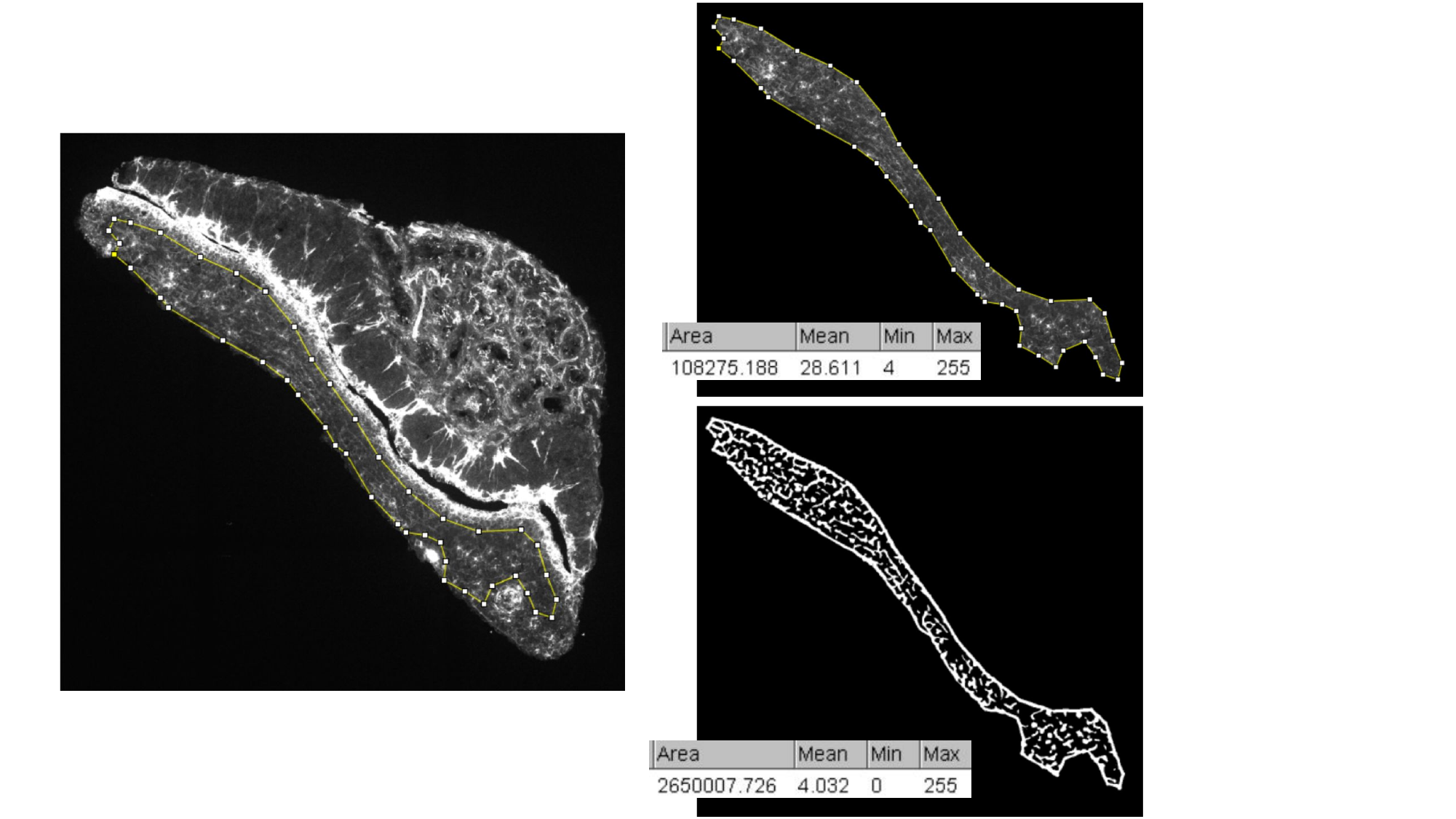

## Slide 9
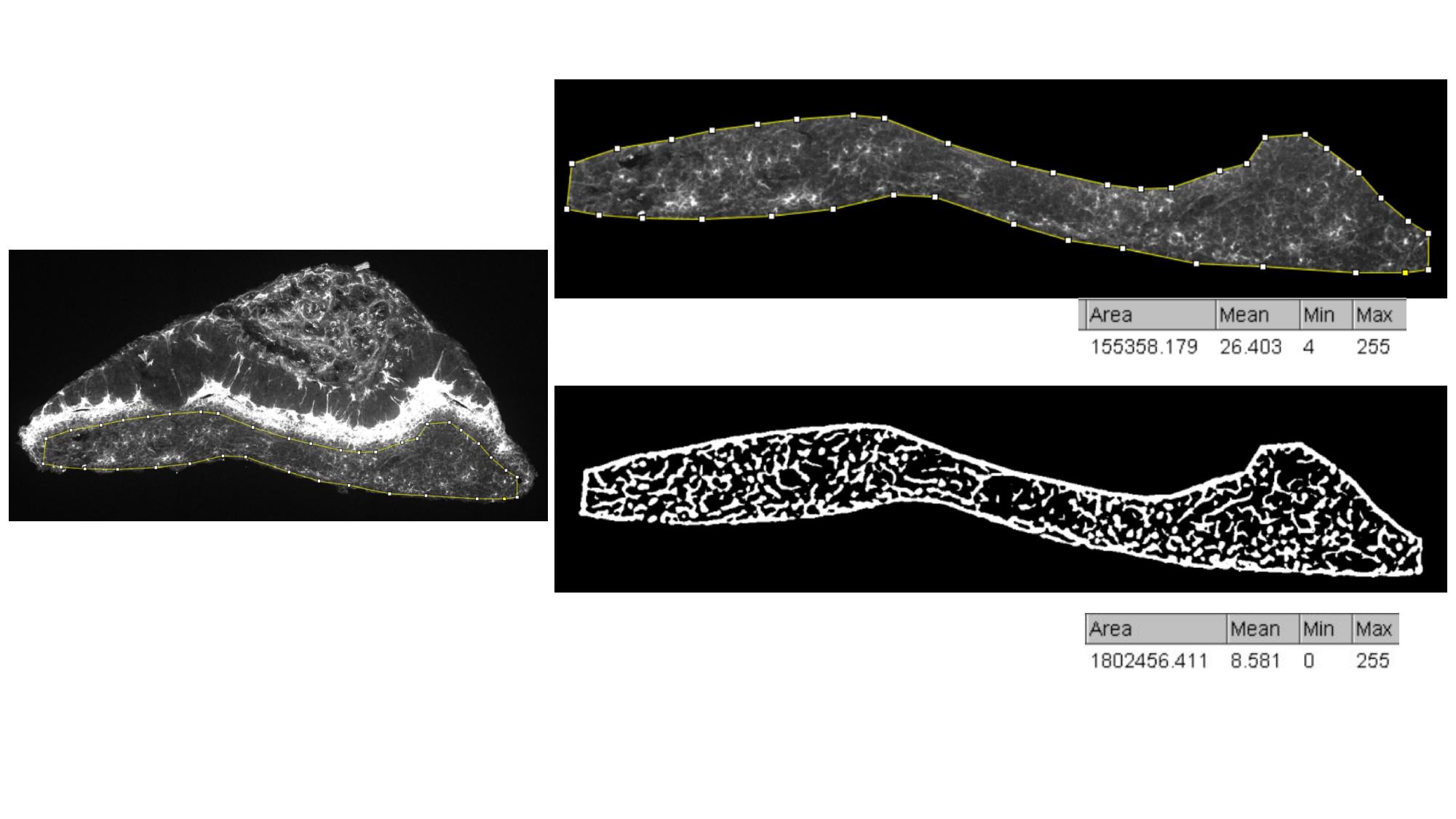

## Slide 10
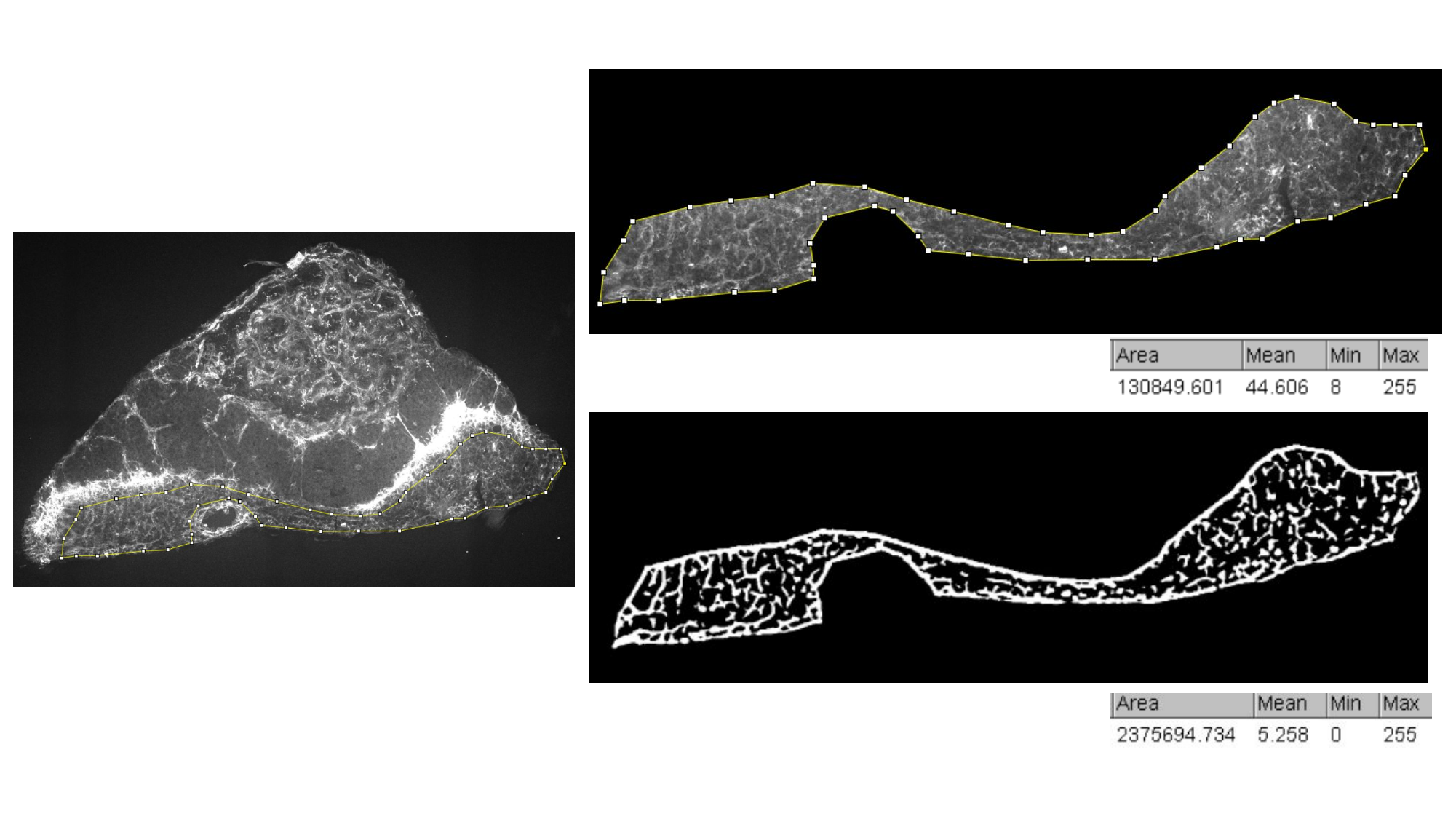

## Slide 11
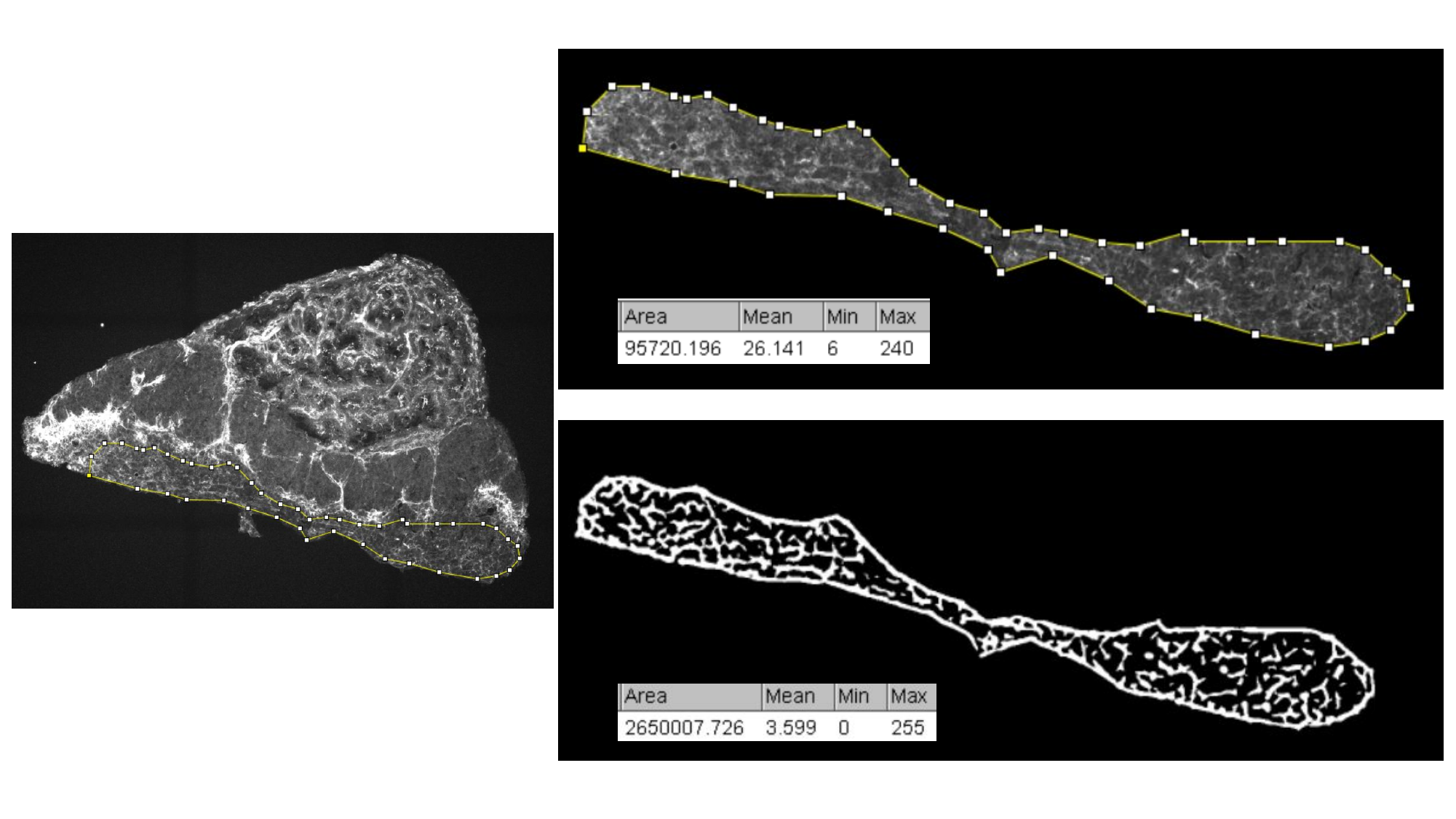

## Slide 12
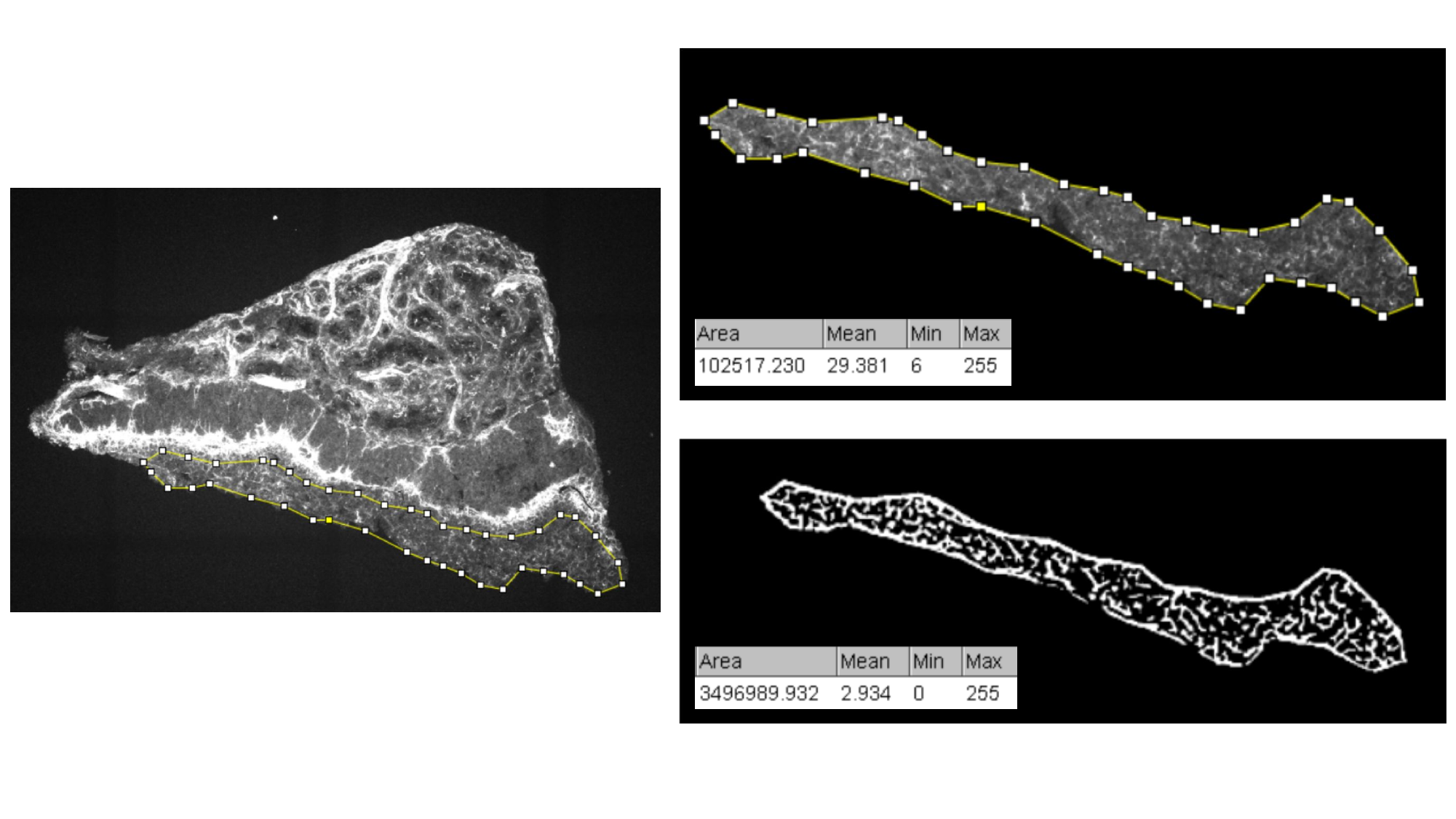

## Slide 13
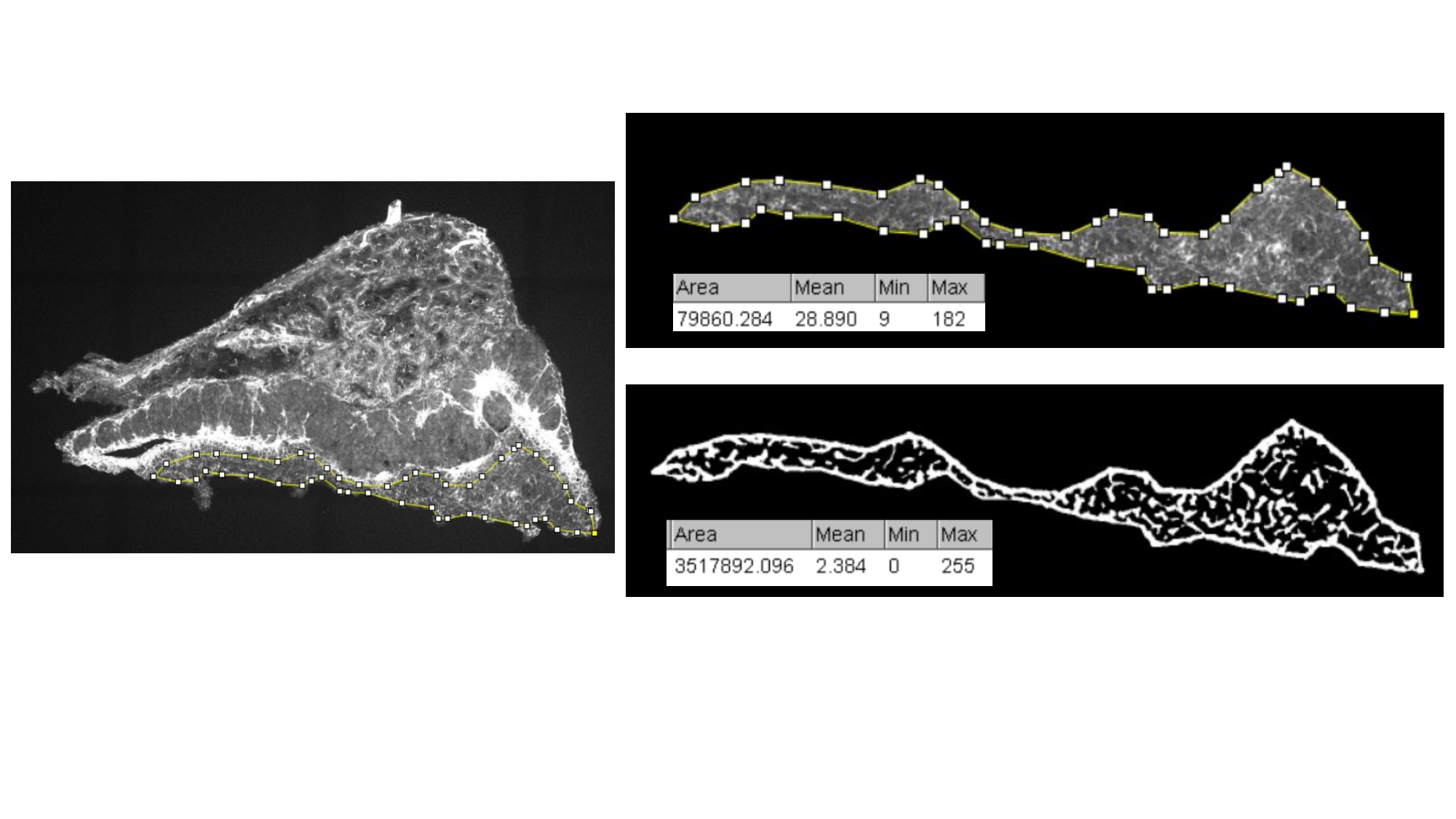

## Slide 14
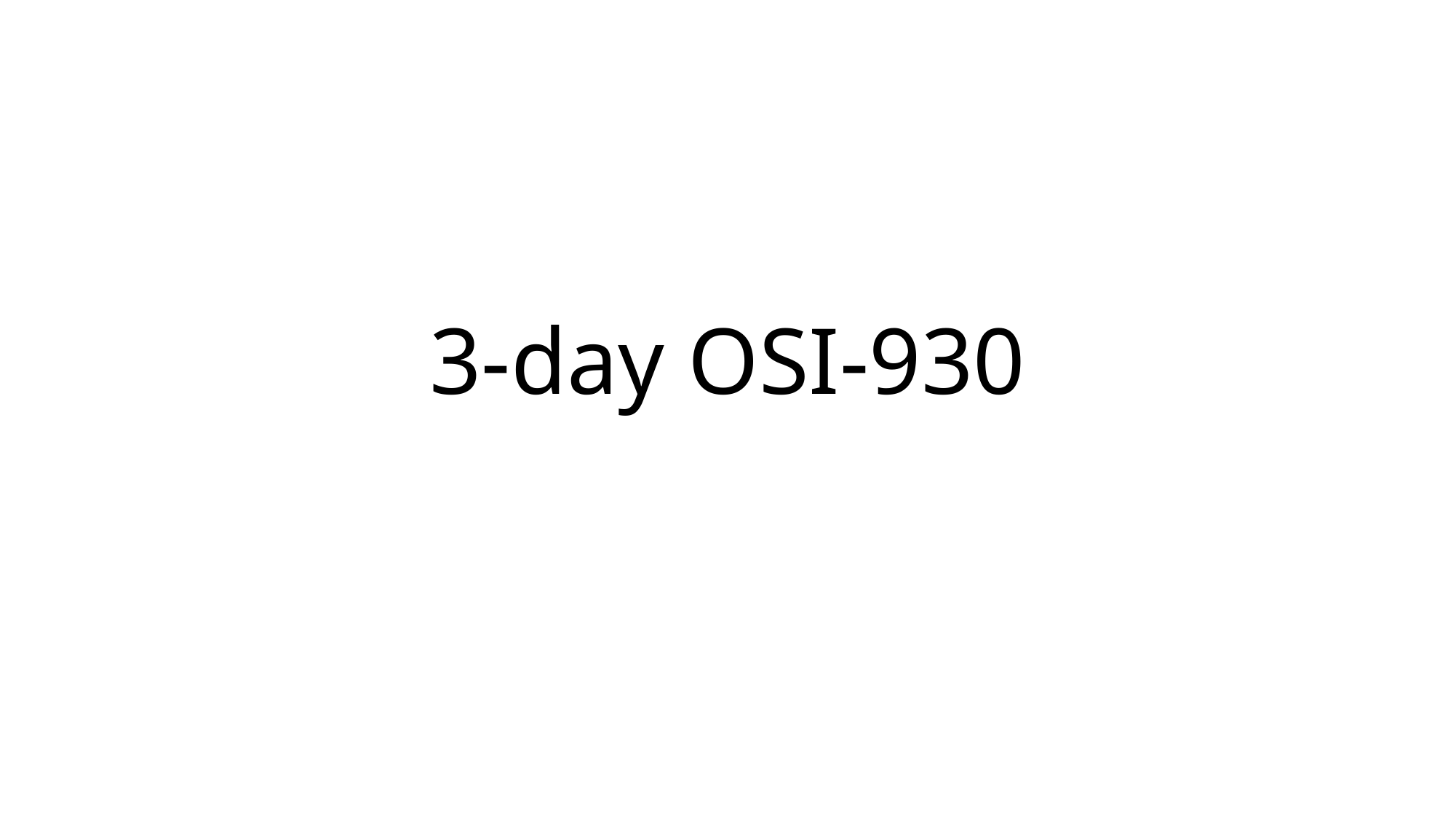

# 3-day OSI-930

## Slide 15
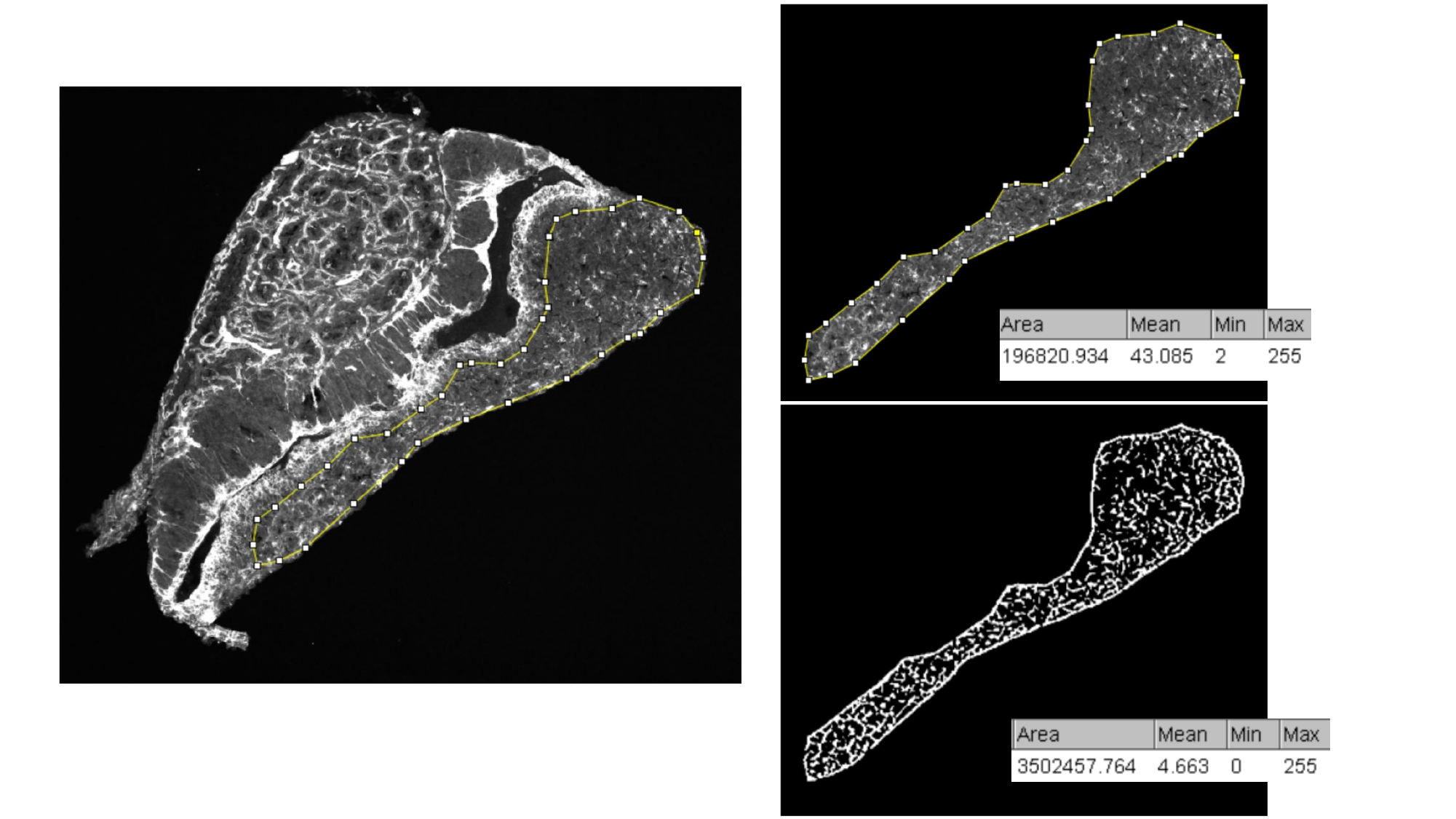

## Slide 16
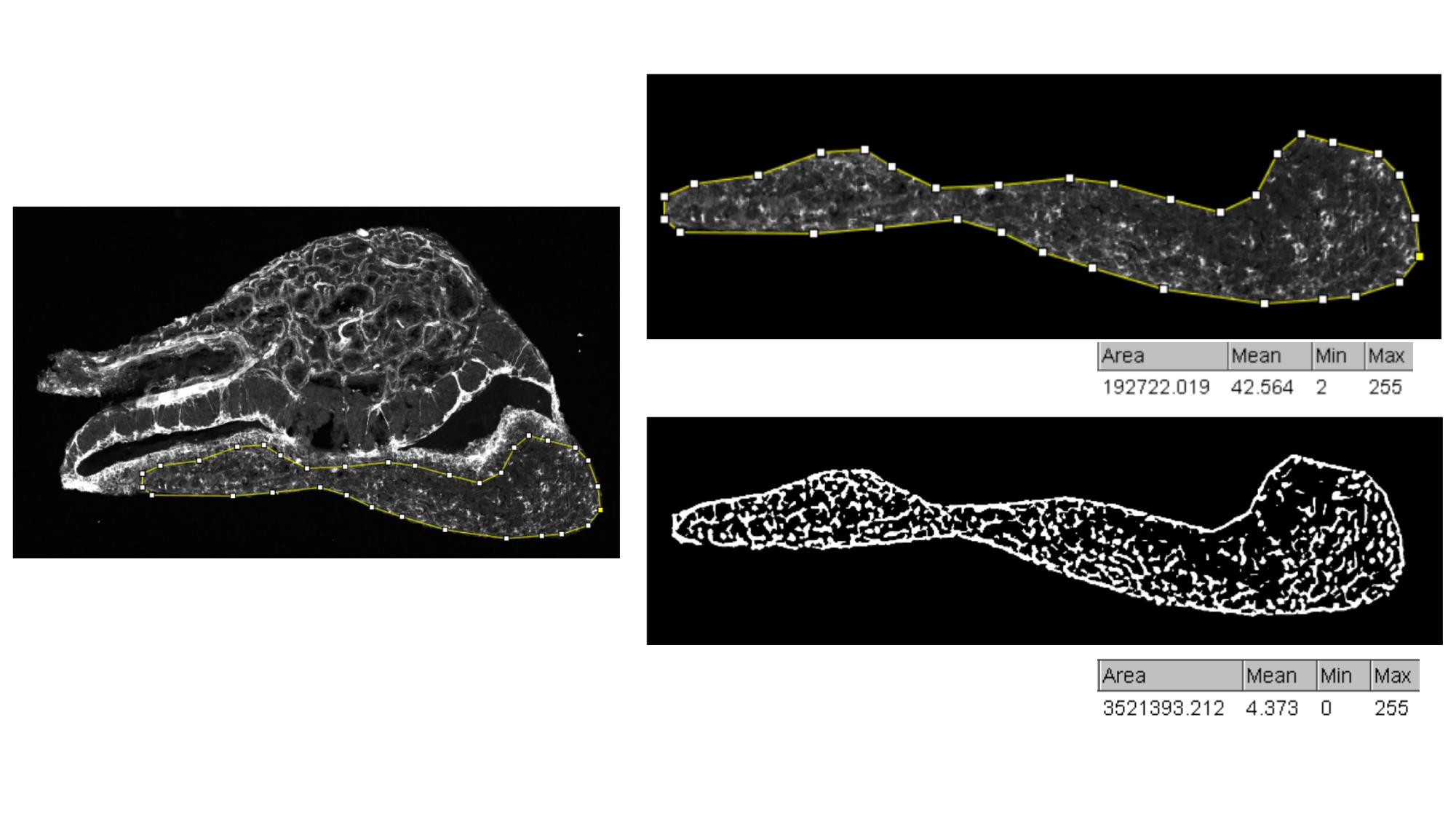

## Slide 17
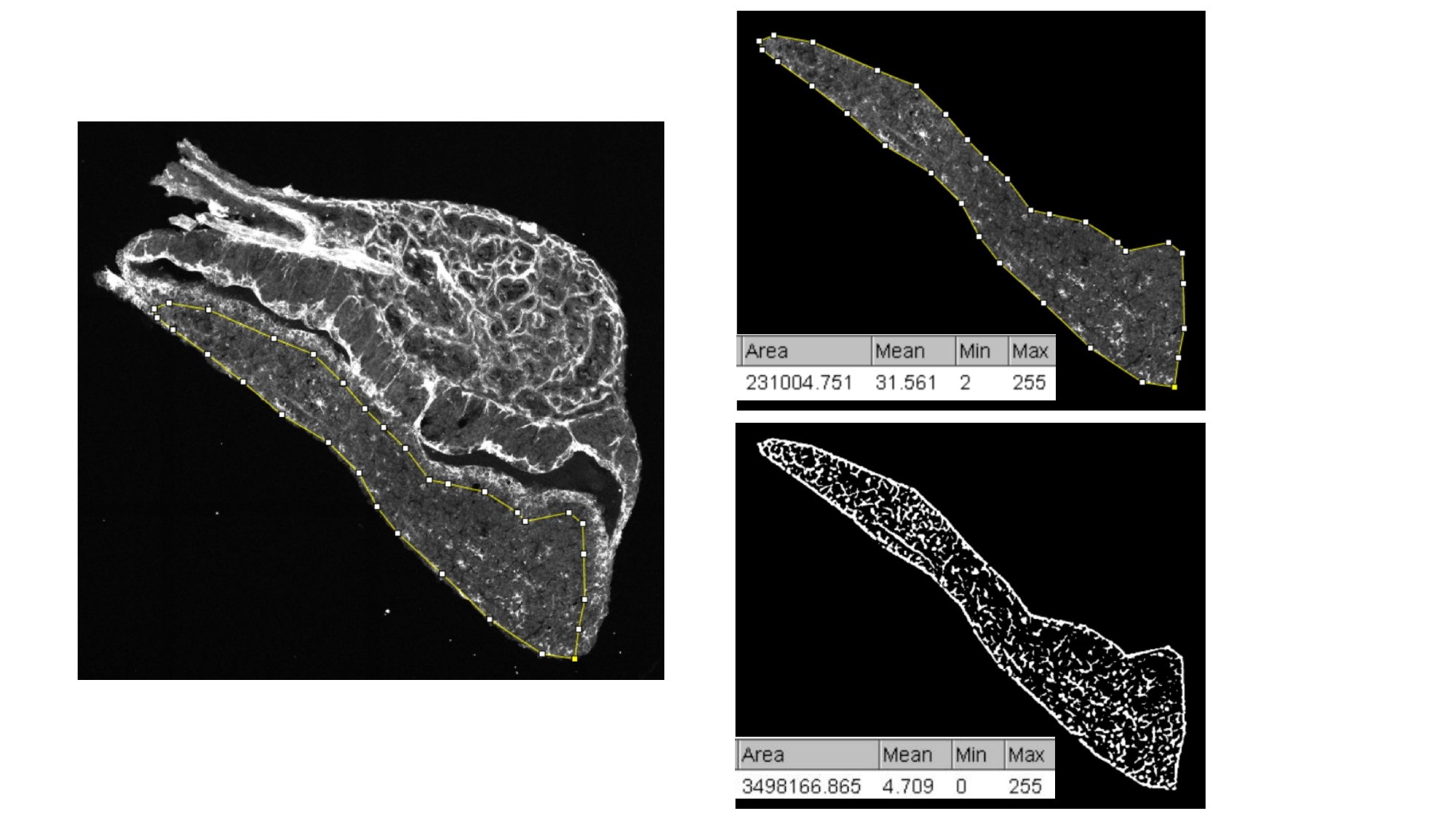

## Slide 18
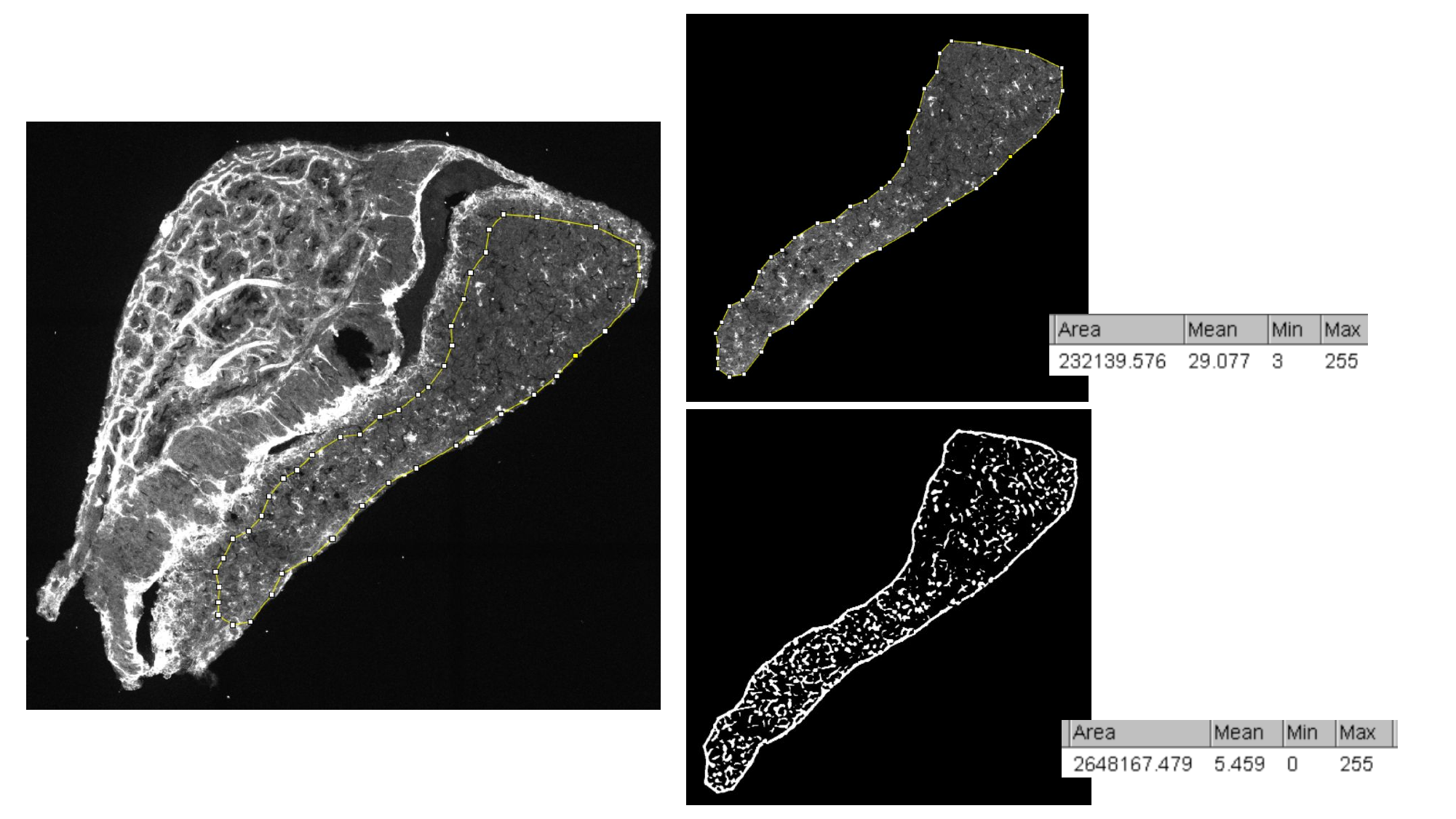

## Slide 19
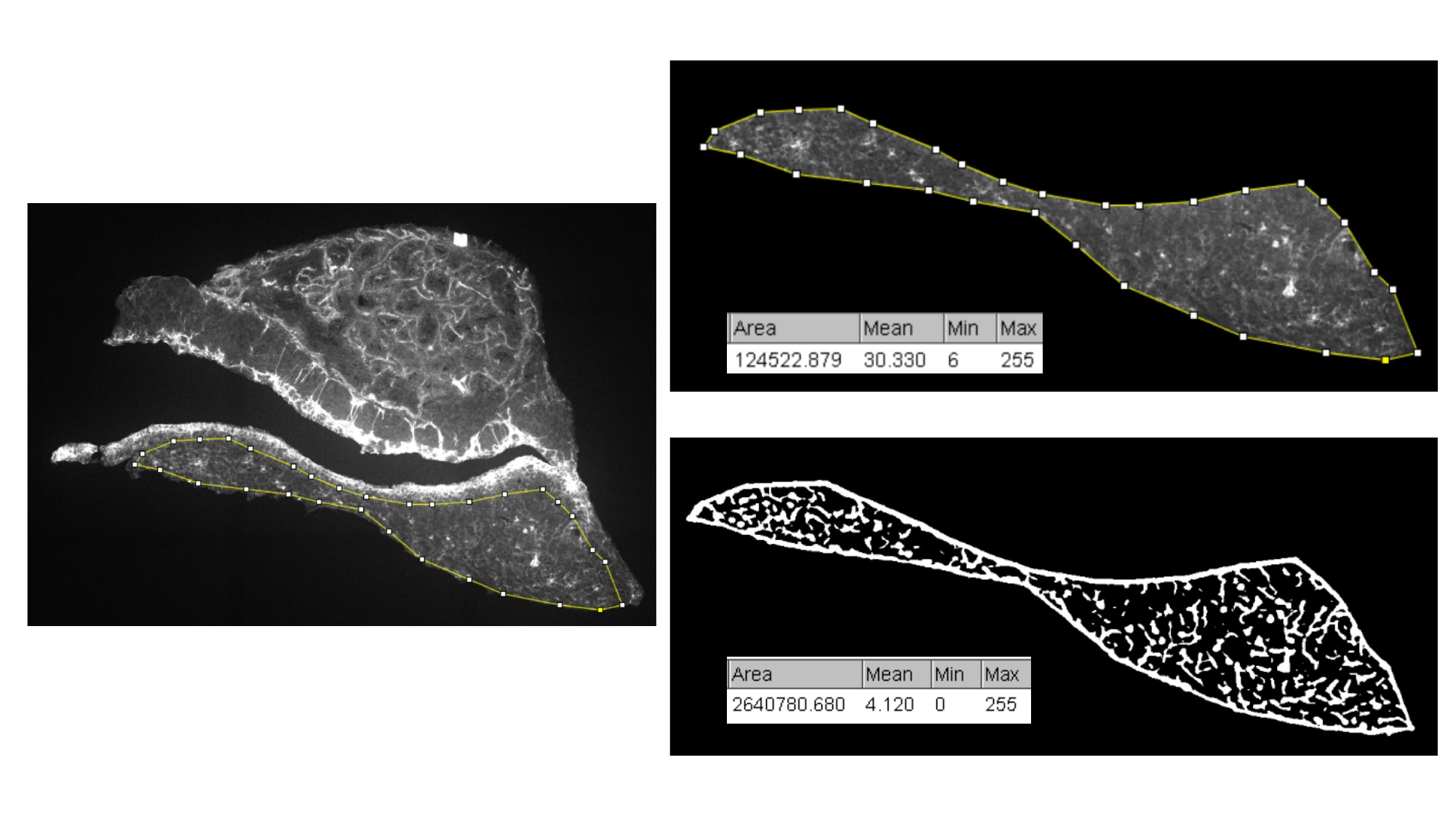

## Slide 20
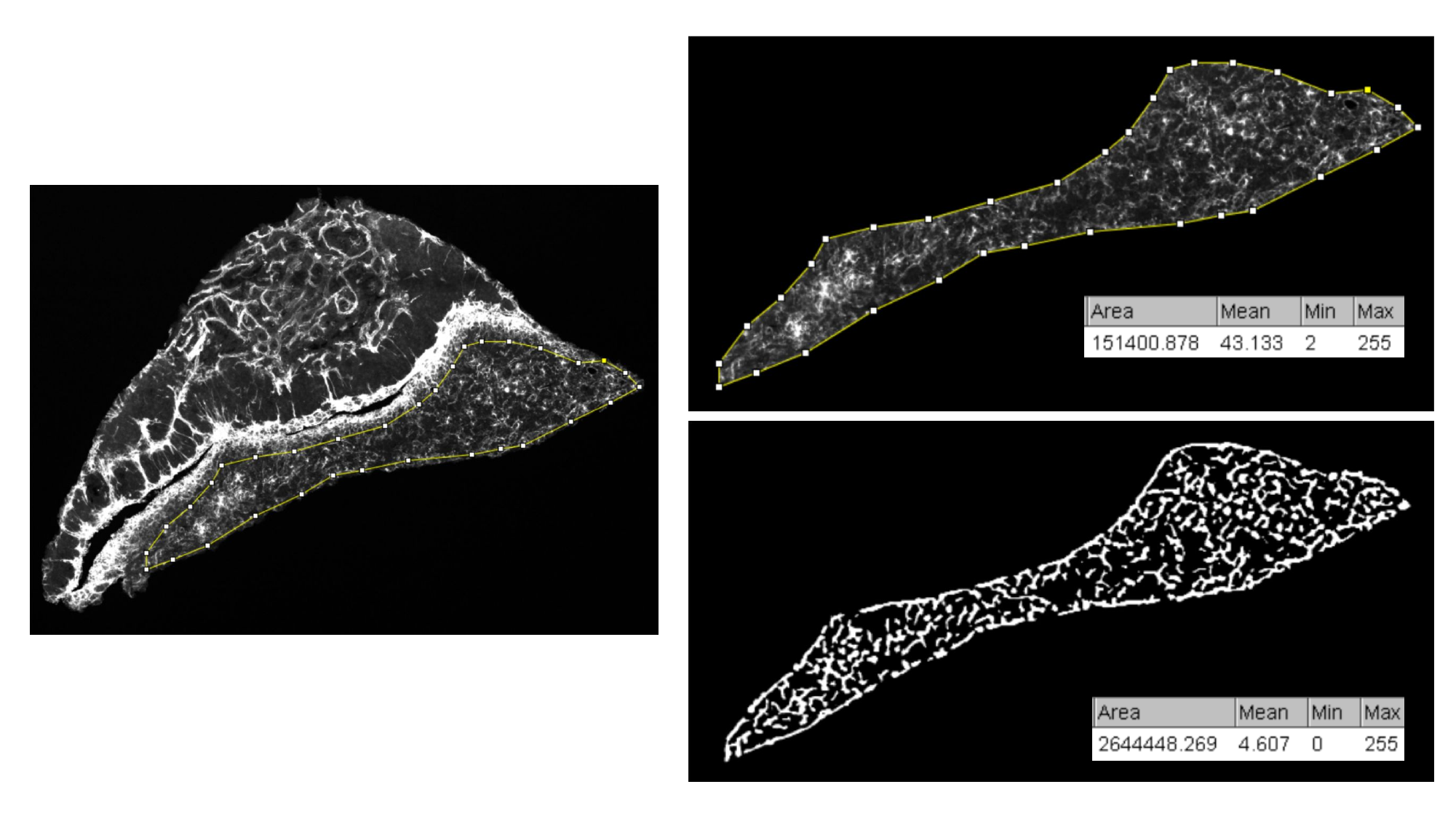

## Slide 21
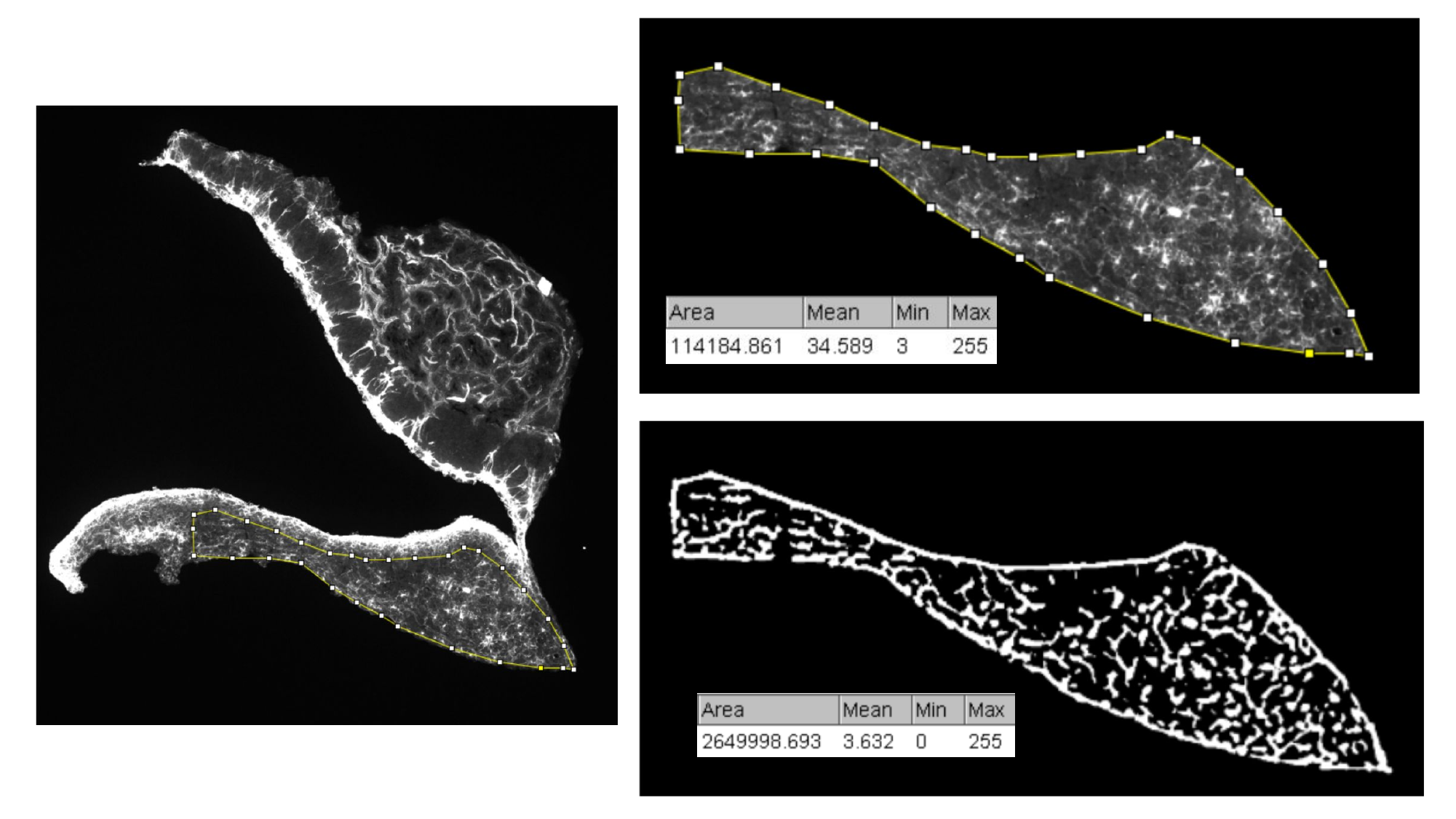

## Slide 22
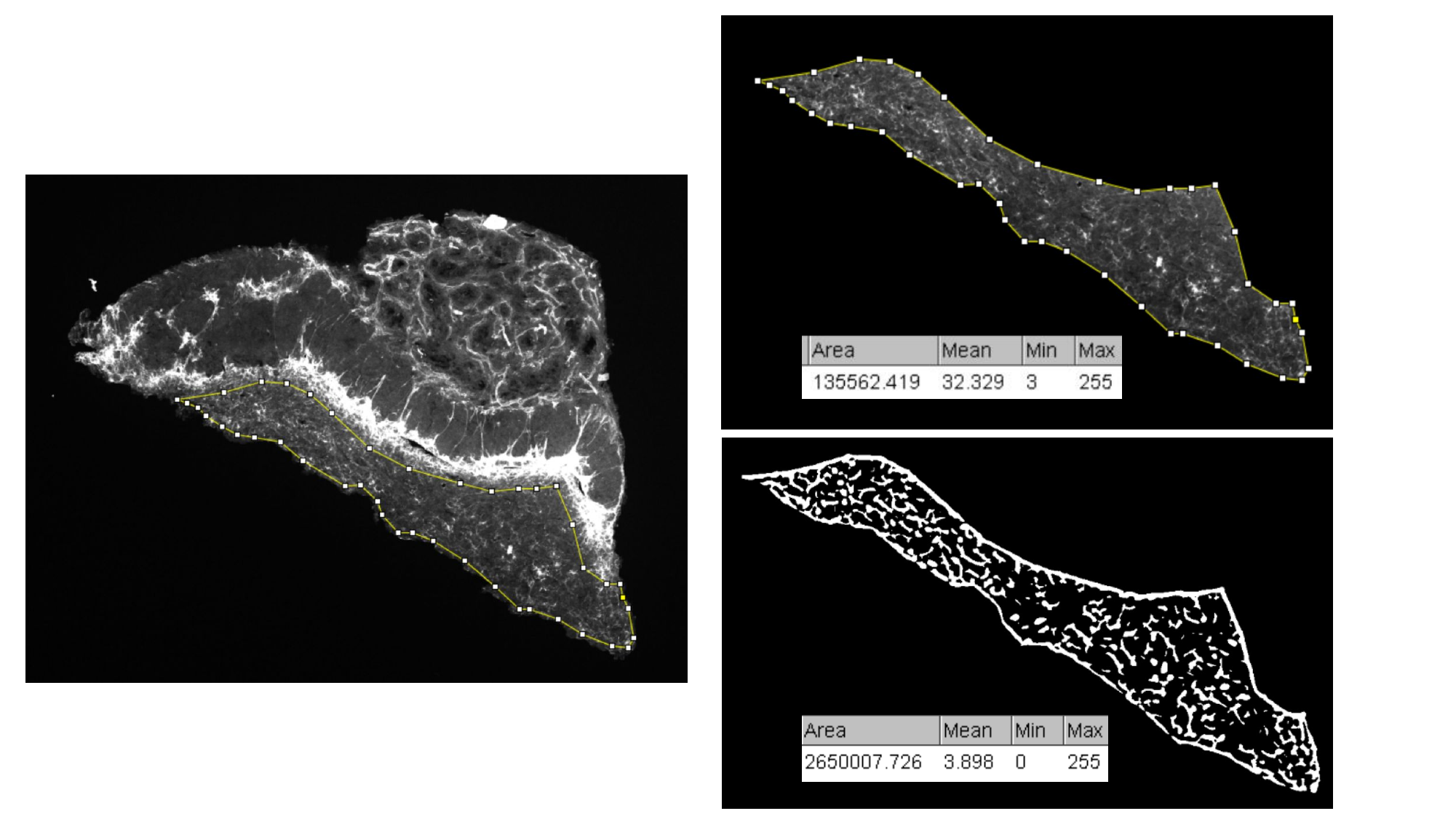

## Slide 23
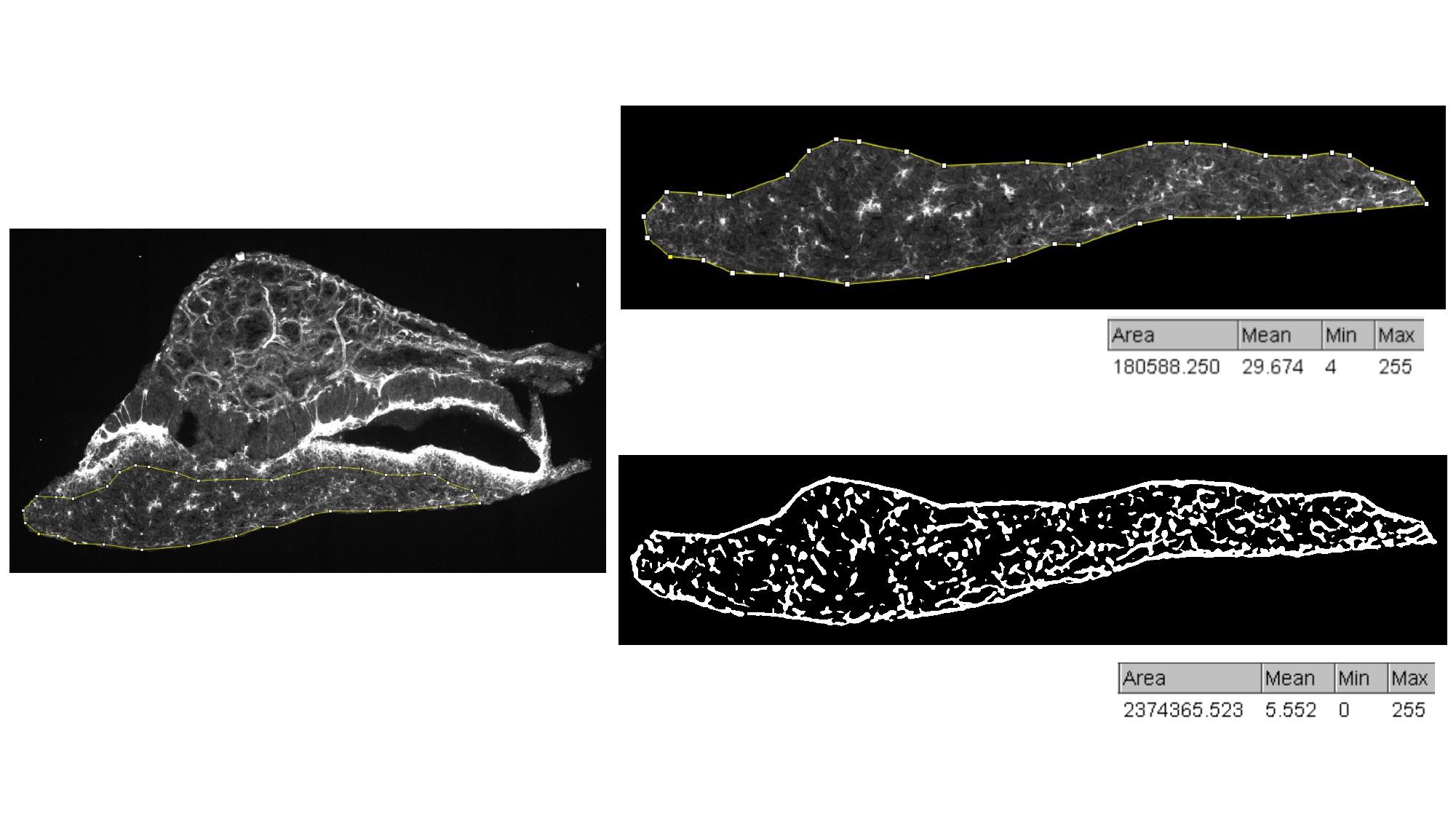

## Slide 24
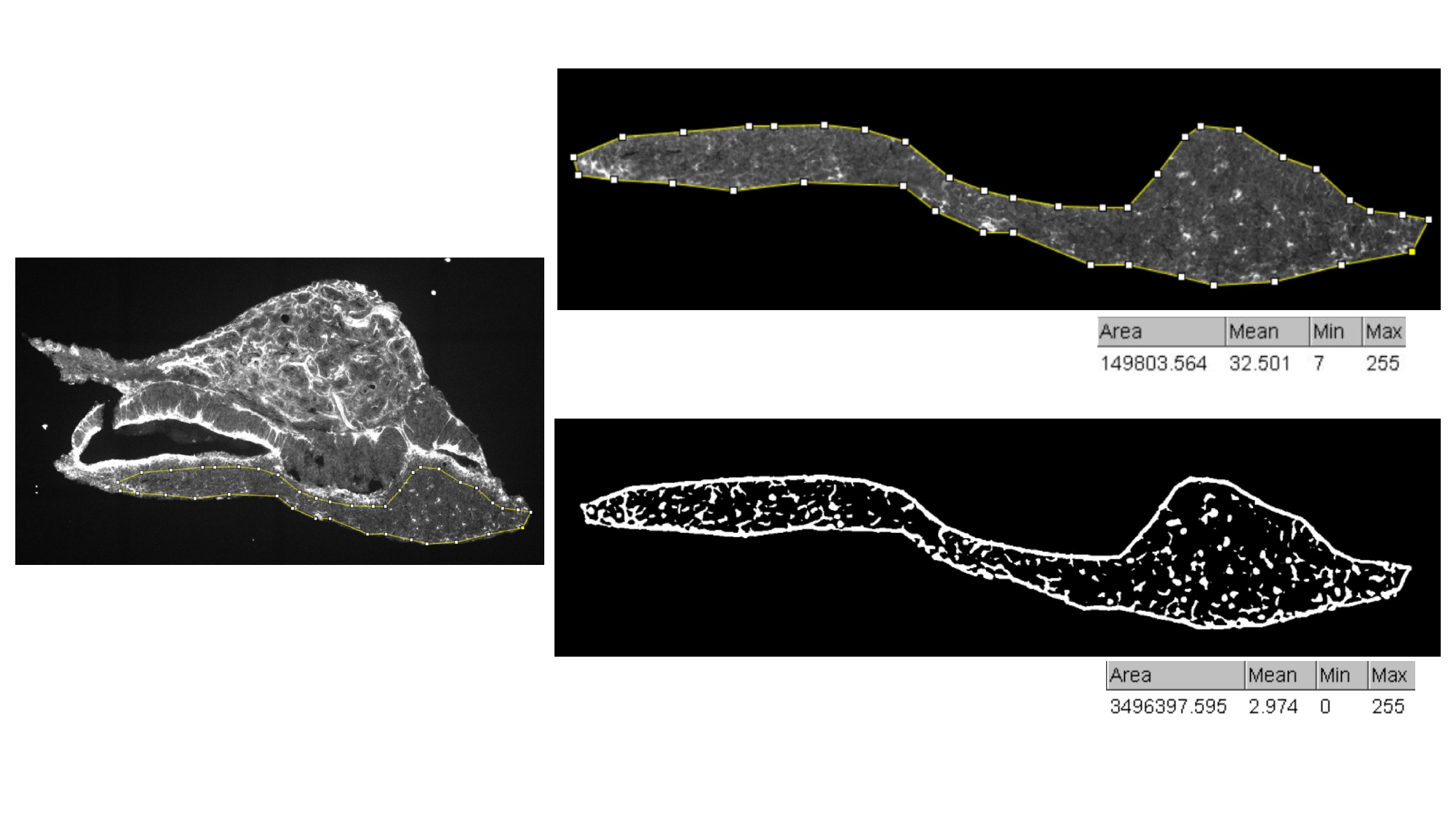

## Slide 25
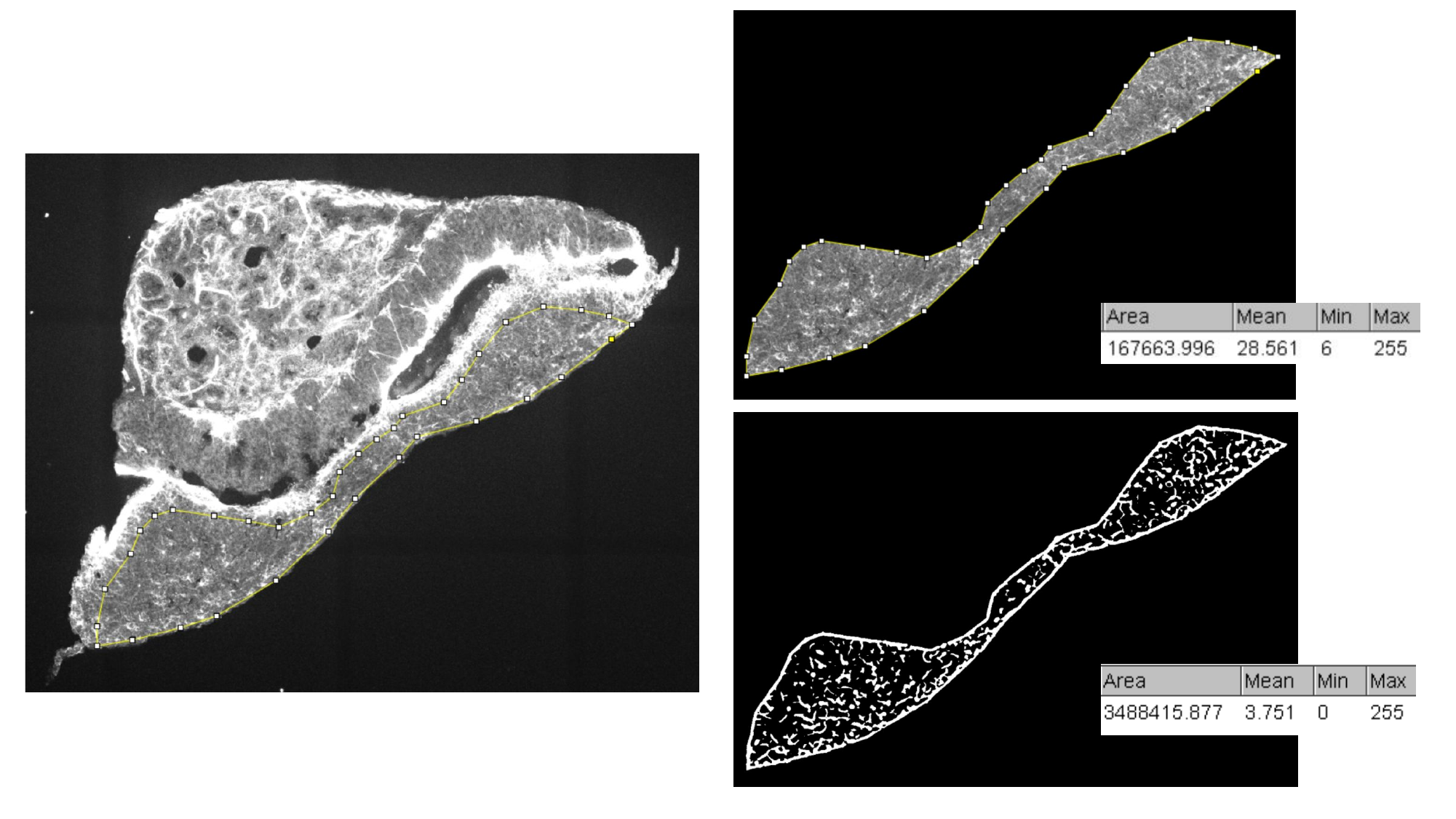

## Slide 26
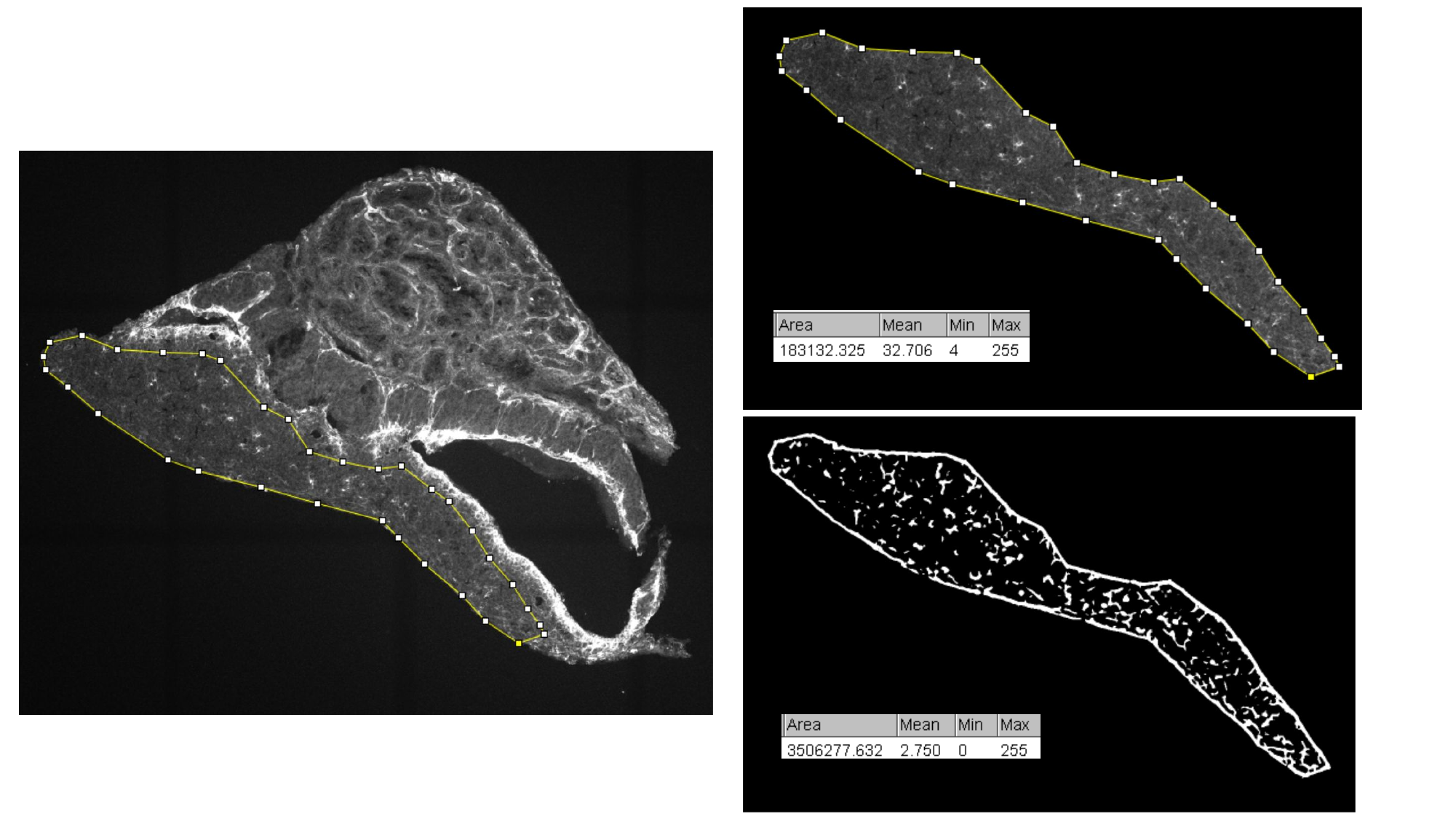

Supplement: S1 File — (PPTX) [file pone.0279634.s001.pptx]
