# Supplementary material for: Inhibition of VEGF receptors induces pituitary apoplexy: An experimental study in mice
Source: PLoS One. 2023 Mar 16;18(3):e0279634. doi: 10.1371/journal.pone.0279634 (PMC10019612; doi:10.1371/journal.pone.0279634)
Supplement: Attachment — Submitted filename: responce to reviewers.docx [file pone.0279634.s002.docx]

**Comments for Journal Requirements (2):**

1. Please provide details on methods of sacrifice.

**Response:** We have revised the Animals section in Materials and method as follows.

“ To harvest tissues, mice were placed under 5% isoflurane until all reflexes were absent and euthanized via cardiac puncture with care to minimize pain. “ (L88)

“Experimental animals C57BL/6N male mice (8-12-week-old, 22-28g) were purchased from Japan SLC (Shizuoka, Japan) and housed under a 12-hour light/dark cycle (8:00–20:00 on, 20:00–8:00 off) with free access to normal autoclaved diet and water. Mouse room temperature was maintained at 23 ºC.” (L74)

“We followed NIH guidelines to treat mice and made every effort to minimize the suffering of the mice and the number of mice used for the experiments. The study was designed and carried out in compliance with the ARRIVE guidelines [15]. Male C57BL/6 mice were randomly divided into six groups with three mice, and injected intraperitoneally with 100 mg/kg body weight of OSI-930 daily for 1, 3, or 6 days. Because of additional experiments, the total number of mice in three-day and six-day OSI-930 treated groups was six and five, respectively. The dosage of OSI-930 was determined based on the study in xenograft models” (L79)

**Comments for Journal Requirements (1):**

1. Please ensure that your manuscript meets PLOS ONE's style requirement.

**Response**: We have reviewed our manuscript to ensure it meet PLOS ONE's style requirement.

2. Please provide additional information regarding the experiments involving animals.

**Response:** We have revised the Animals section in Materials and method as follows.

“ To harvest tissues, mice were placed under 5% isoflurane until all reflexes were absent and euthanized via cardiac puncture with care to minimize pain. “ (L88)

“Experimental animals C57BL/6N male mice (8-12-week-old, 22-28g) were purchased from Japan SLC (Shizuoka, Japan) and housed under a 12-hour light/dark cycle (8:00–20:00 on, 20:00–8:00 off) with free access to normal autoclaved diet and water. Mouse room temperature was maintained at 23 ºC.” (L74)

“We followed NIH guidelines to treat mice and made every effort to minimize the suffering of the mice and the number of mice used for the experiments. The study was designed and carried out in compliance with the ARRIVE guidelines [15]. Male C57BL/6 mice were randomly divided into six groups with three mice, and injected intraperitoneally with 100 mg/kg body weight of OSI-930 daily for 1, 3, or 6 days. Because of additional experiments, the total number of mice in three-day and six-day OSI-930 treated groups was six and five, respectively. The dosage of OSI-930 was determined based on the study in xenograft models” (L79)

3. All PLOS journals require that the minimal data set be made fully available.

**Response:** We have uploaded the raw data for the analysis as supporting information file.

4. PLOS does not permit references to inaccessible data.

**Response:** We have removed from Results section the results that do not display any data. All results now properly display the data on which they are based.

**Comments for Reviewers:**

**Reviewer: 1**

Major comments:

“It is not well known, but I do that GS-IB4 react the folliculo-stellated cells and marginal cells in the intermediate lobe in mouse anterior pituitary.”

**Response**: We have newly harvested the pituitary gland from mice, and performed double staining with ILB4 and anti-PECAM1 antibody, and double staining with ILB4 and anti-PLVAP antibody. The results suggest that ILB4 staining is positive for structures other than vessels in the MCL, but can also be used to some extent in order to analyze pituitary vessels, although there are some limitations. We have added Fig 3, and added sentences as follows.

“To examine whether this isolectin B4-positive structures are blood vessels or not, we performed immunohistochemical analysis of PECAM1 (CD31, Figs 3A-H) and PLVAP (Figs 3I-P). Isolectin B4 staining were observed around PECAM1-positive or PLVAP-positive blood vessels in the MCL (Figs 3A-P), suggesting isolectin B4-positive structures are blood vessels.” (L168)

“Fig 3. Comparison of isolectin B4 staining with PECAM1 and PLVAP immunostaining. Representative confocal immunofluorescence sagittal images with orthogonal views. Boxed regions in A-D and I-L are enlarged in E-H and M-P, respectively. Arrows and arrow heads indicate the intersection points of the XZ and YZ planes. (A-H) The pattern of isolectin B4 staining (red) was extremely similar to that of PECAM1 immunostaining (green), including the surrounding area of Rathke's cleft. (I-P) A significant number of cells positive for isolectin B4 staining were positive for PLVAP. Bars, 200 µm. AL, anterior lobe; IL, intermediate lobe; PL, posterior lobe.” (L192)

Minor comments:

“Authors used sections at 30 μm thickness for H&E staining? It is not fine data (Fig. 1A-F). Authors should use 6-10 μm thickness sections.”

**Response**: We have newly harvested the pituitary gland from mice, made sections at 10 μm thickness, and performed H&E staining. We have revised Fig 1, and added sentences as follows.

“For H&E staining, the pituitary glands were fixed in formalin for 2 days and sections were made at 10 μm thickness.” (L96)

“Page 2 line 26: Two of three three-day?”

**Response**: We have revised the sentence as follows. The number of mice analyzed has increased with additional experiments.

“Four of six mice treated for three days and all of five mice treated for six days exhibited hemorrhage in the pituitary gland.” (L26)

“Page 2 line 31: The expression of anti-?”
**Response**: We have corrected the sentence as follows.

“Few blood vessels around Rathke’s cleft of the intermediate lobe express VE-cadherin and are covered with platelet-derived growth factor receptor-β (PDGFR-β)-positive cells” (L31)

“Page 6 line 90: Lica?”

**Response**: We have corrected the sentence as follows.

“confocal laser scanning microscopy (Leica).” (L108)

**Reviewer: 2**

Major comments.
1. Since PECAM1 and PLVAP/PV-1 are common markers for pituitary endothelial cells, so you should also use that antibody to confirm that the isolectin B4-positive cells around the MCL are endothelial cells.

**Response**: We have newly harvested the pituitary gland from mice, and performed double staining with ILB4 and anti-PECAM1 antibody, and double staining with ILB4 and anti-PLVAP antibody. The results suggest that ILB4 staining is positive for structures other than vessels in the MCL, but can also be used to some extent in order to analyze pituitary vessels, although there are some limitations. We have added Fig 3, and added sentences as follows.

“To examine whether this isolectin B4-positive structures are blood vessels or not, we performed immunohistochemical analysis of PECAM1 (CD31, Figs 3A-H) and PLVAP (Figs 3I-P). Isolectin B4 staining were observed around PECAM1-positive or PLVAP-positive blood vessels in the MCL (Figs 3A-P), suggesting isolectin B4-positive structures are blood vessels.” (L168)

“Fig 3. Comparison of isolectin B4 staining with PECAM1 and PLVAP immunostaining. Representative confocal immunofluorescence sagittal images with orthogonal views. Boxed regions in A-D and I-L are enlarged in E-H and M-P, respectively. Arrows and arrow heads indicate the intersection points of the XZ and YZ planes. (A-H) The pattern of isolectin B4 staining (red) was extremely similar to that of PECAM1 immunostaining (green), including the surrounding area of Rathke's cleft. (I-P) A significant number of cells positive for isolectin B4 staining were positive for PLVAP. Bars, 200 µm. AL, anterior lobe; IL, intermediate lobe; PL, posterior lobe.” (L192)

2. Observation of the anterior lobe structure is easy to observe in the frontal section but not sagittal section, you should analyze changes in the vascular structure of the anterior lobe.

**Response**: Although there is a limit to the number of mice that can be prepared for additional experiments, we administered new doses of OSI to as many mice as possible, harvested the pituitary gland, and, whenever possible, created a new coronal section and analyzed the anterior lobe. We have revised Fig 2 and 6, and revised sentences as follows.

“**Fig 2. VEGFR inhibition disrupts pituitary vasculature. (A-F)** Representative frontal sectional images of pituitaries labelled by isolectin B4 staining in control (A and D), three-day OSI-930-treated (B and E), and six-day OSI-930-treated (C and F) groups. Boxed regions in A-C are enlarged in D-F, respectively. **(G–****L)** Representative sagittal images of pituitaries labelled by isolectin B4 staining in control (G and J), three-day OSI-930-treated (H and K), and six-day OSI-930-treated (I and L) groups. Boxed regions in G-I are enlarged in J-L, respectively. White dots indicate capillary plexus with a palisade-fashion arrangement in the intermediate lobe. In OSI-930-treated groups, hematoma cavities were observed around Rathke’s cleft (arrows; B**,** C, K, and L), and capillary networks in the intermediate lobe were decreased compared to control (dots: J-L). In addition, capillaries around Rathke’s cleft were diminished compared to control. **(M-P)** Quantitation of vascular area, branches and branching points in the anterior lobe, and vascular networks in the intermediate lobe. The vascular are (M), branches (N) and branching points (O) were decreased in the anterior lobe, and the number of capillary plexus with a palisade-fashion arrangement was decreased in the intermediate lobe (P) in a treatment-day dependent manner (* P < 0.05, t-test). Bars, 200 µm. AL, anterior lobe; IL, intermediate lobe; PL, posterior lobe; C, control.” (L174)

“In the anterior lobe, the vascular coverage, vascular branches and branching points were decreased in a treatment-day dependent manner (Figs 2M-O).” (L202)

“VE-cadherin was expressed in vessels of anterior and posterior lobes (Figs 6A-F). However, Few blood vessels of the intermediate lobes express VE-cadherin. As with pericytes, distribution of VE-cadherin was sparse in capillaries around Rathle’s cleft, where hemorrhagic change was frequently observed after OSI-930 administration (Figs 6G-M). Taken together, undeveloped pericytes and VE-cadherin may have influenced the susceptibility to bleeding in capillaries around Rathke’s cleft. **Fig 6. Few blood vessels of the intermediate lobes express VE-cadherin.** **(A-M)** Representative frontal sectional images of pituitaries stained with isolectin B4 (red) and anti-VE-cadherin antibody (green).” (L240)

3. You should clarify the change in the expression level of marker genes (Pecam1, Plvap, Flt1, Flk1, etc.) including VE-cadherin (Cdh5) in the pituitary by qPCR.

**Response**: We have newly analyzed vascular markers including Pecam1, Plvap, Flt1, Flk1, and VE-cadherin (Cdh5) in the pituitary gland by qRT-PCR. We have added Fig 4 and added sentences as follows.

**“Quantitative real time RT-PCR analysis**

Total RNA was extracted from the pituitary gland using RNA easy Mini kit (Qiagen, Hilden, Germany). Complementary DNA was obtained using a PrimeScript 1st strand cDNA synthesis kit (Takara, Shiga, Japan). Gene expressions were quantified using TB Green　Premix Ex Taq II　(Tli RNaseH Plus) (Takara, Shiga, Japan). Quantitative real-time PCR was performed on a LightCycler 480 system (Roche, Basel, Switzerland). *Actb* was used as a control (Mouse Housekeeping Gene Primer Set (Takara, Shiga, Japan)). The following primers were used for qPCR: Pecam1 forward: tcggcagacaagatgctcctggctc, Pecam1 reverse: cagggtcagttgctgcccattcatcacc, *Plvap* forward: gctatcatcctgagcgagaagcagtgcc, *Plvap* reverse: tgccttctccttggccacctccatc, *Flt1* forward: ctgcgaagccaccgtcaacggg, *Flt1* reverse: gtggcggtgcagttgaggacaagag, *Flk1* forward: cgtaccgggacgtcgacatagcctc, *Flk1* reverse: ggtgatgtacacgatgccatgctggtc, *VE-cadherin (Cdh5)* forward: ctgccctcattgtggacaagaacaccaac, *VE-cadherin (Cdh5)* reverse: gacatctctggcacagatgcgttgaatacctg.” (L123)

“These OSI effects on blood vessels were also detected by the change in the expression levels of VEGFR1 and VEGFR2 but not of PECAM1, PLVAP, and VE-cadherin (Fig 4).” (L203)

**“Fig 4. Quantitative real-time RT-PCR analysis of vascular markers.** Expression levels of PECAM1 (A), PLVAP (B), VEGFR1 (C), VEGFR2 (D), and VE-cadherin (E) were analyzed. *Actb* was used as a control (* P<0.05, t-test).” (L212)

“In our study, the expression of VEGFR1 was decreased after OSI-930 administration. This may reflect hormonal cell destruction by pituitary apoplexy. The expression of VEGFR2 was also decreased. This may reflect the vascular regression in pituitaries.“ (L283)

4. Pituitary apoplexy may be due to changes of endothelial fenestration with OSI-930 treatment. It may be necessary to observe the ultrafine structure of endothelial cells using an electron microscopy to reveal the fenestra structure in the pituitary of mouse with OSI-930 treatment.

**Response**: An electron microscopic analysis was attempted, but technically could not be performed. Therefore, the text has been revised as follows to clarify the limitations of this paper. We have added sentences as follows.

“The capillaries distributed in the pituitary gland are characteristically rich in fenestrations [26, 27].　It has been reported that VEGFR inhibitor administration reduced endothelial fenestrations in thyroid perifollicular capillaries, renal glomerular capillaries, and capillaries in islet-cell tumors of RIP-Tag2 transgenic mice [7, 28]. To date, no studies have been reported on fenestration changes after VEGFR inhibitor administration in pituitary capillaries. Due to lack of access to an electron microscopy, we could not examine ultrastructural changes in pituitary capillary endothelial fenestrations. Pituitary apoplexy may be caused due to changes of endothelial fenestration with OSI-930 treatment.” (L295)

Minor comments.
1． Separate the figure legend from the text and summarize it.

**Response**: Sorry, but PLOS ONE submissions requirements say that Each figure caption should appear directly after the paragraph in which they are first cited.

2． Please correct line 90 Lica to Leica.

**Response**: We have corrected it. (L108)

3． Please correct line 229 VRGF to VEGF.

**Response**: We have corrected it. (L309)

4． The notation of AL and PL in Figure 1A is reversed.

**Response**: We have corrected it. (Fig 1)
