# Supplementary material for: Inhibition of VEGF receptors induces pituitary apoplexy: An experimental study in mice
Source: PLoS One. 2023 Mar 16;18(3):e0279634. doi: 10.1371/journal.pone.0279634 (PMC10019612; doi:10.1371/journal.pone.0279634)
Supplement: Attachment — Submitted filename: Response to Reviewers.docx [file pone.0279634.s003.docx]

**Comments for Journal Requirements:**

Please review your reference list to ensure that it is complete and correct. If you have cited papers that have been retracted, please include the rationale for doing so in the manuscript text, or remove these references and replace them with relevant current references.  Any changes to the reference list should be mentioned in the rebuttal letter that accompanies your revised manuscript.

**Response:** Three authors (MH, KT, and YS) independently and carefully checked the reference list to ensure that retracted papers were not cited. Our manuscript did not cite retracted papers. Because the description of reference 15 was inadequate, we have corrected it as follows.

“15. Percie du Sert N, Hurst V, Ahluwalia A, Alam S, Avey MT, Baker M, et al. The ARRIVE guidelines 2.0: Updated guidelines for reporting animal research. PLoS Biol. 2020;18(7):e3000410. Epub 2020/07/15. doi: 10.1371/journal.pbio.3000410. PubMed PMID: 32663219; PubMed Central PMCID: PMCPMC7360023.“ (L380)

**Comments for Reviewers:**

**Reviewer #2:**

1. In materials and methods, you must indicate the city and country of vendors of the reagents and equipment. Also, please add the product numbers of all antibodies.

**Response**: We have revised the materials and methods section as follows.

“mouse anti-platelet endothelial cell adhesion molecule 1 (PECAM1) / CD31 (1:10; clone 390, 102402, BioLegend, SanDiego, USA), mouse anti-plasmalemma vesicle associated protein (PLVAP; 1:100; ab27853, Abcam, Cambridge, UK), mouse anti-platelet-derived growth factor receptor-β (PDGFR-β)/CD140b (1:100; 136002, BioLegend, SanDiego, USA), and rat anti-VE-cadherin/CD144 (1:100; clone 11D4.1, 555289, BD, NJ, USA) [16]. Alexa Fluor 568-conjugated isolectin GS-IB4 from *Griffonia simplicifolia* (1:100; I21412, Thermo Fisher Scientific, MA, USA) was used to label endothelial cells. Briefly, cryosections were incubated with primary antibodies for 24 h at 4 ℃, and then with secondary antibodies for 1 h at room temperature. Donkey anti-species IgG conjugated with Alexa 488 (A21206, A21208, Thermo Fisher Scientific, MA, USA) was used for a secondary antibody. Samples were then treated with DAPI. Sections were analyzed with Leica SP8 confocal laser scanning microscopy (Leica, Wetzlar, Germany). “ (L99)

2. In references, please delete the "conflict of interest" part after line 382 because it is unnecessary.

**Response**: We have deleted the "conflict of interest" part in reference 15 as follows.

“15. Percie du Sert N, Hurst V, Ahluwalia A, Alam S, Avey MT, Baker M, et al. The ARRIVE guidelines 2.0: Updated guidelines for reporting animal research. PLoS Biol. 2020;18(7):e3000410. Epub 2020/07/15. doi: 10.1371/journal.pbio.3000410. PubMed PMID: 32663219; PubMed Central PMCID: PMCPMC7360023.“ (L380)
