# Supplementary material for: Inhibition of VEGF receptors induces pituitary apoplexy: An experimental study in mice
Source: PLoS One. 2023 Mar 16;18(3):e0279634. doi: 10.1371/journal.pone.0279634 (PMC10019612; doi:10.1371/journal.pone.0279634)
Supplement: Attachment — Submitted filename: Response to Reviewers.docx [file pone.0279634.s005.docx]

**Comments for Journal Requirements:**

**Journal Requirements:**

“Please review your reference list to ensure that it is complete and correct. If you have cited papers that have been retracted, please include the rationale for doing so in the manuscript text, or remove these references and replace them with relevant current references. Any changes to the reference list should be mentioned in the rebuttal letter that accompanies your revised manuscript. If you need to cite a retracted article, indicate the article’s retracted status in the References list and also include a citation and full reference for the retraction notice. “

**Response:** The link between DOI numbers and articles was inadequate. Therefore, we have corrected this problem, and proper linking is now possible. In addition, we have again checked the reference list to ensure that retracted papers were not cited. Our manuscript did not cite retracted papers.
